# Functional innovation promotes diversification of form in the evolution of an ultrafast trap-jaw mechanism in ants

**DOI:** 10.1371/journal.pbio.3001031

**Published:** 2021-03-02

**Authors:** Douglas B. Booher, Joshua C. Gibson, Cong Liu, John T. Longino, Brian L. Fisher, Milan Janda, Nitish Narula, Evropi Toulkeridou, Alexander S. Mikheyev, Andrew V. Suarez, Evan P. Economo

**Affiliations:** 1 Biodiversity and Biocomplexity Unit, Okinawa Institute of Science and Technology Graduate University, Onna, Japan; 2 Department of Ecology and Evolution, University of California-Los Angeles, Los Angeles, California, United States of America; 3 Field Museum of Natural History, Chicago, Illinois, United States of America; 4 Georgia Museum of Natural History, Athens, Georgia, United States of America; 5 Beckman Institute for Advanced Science and Technology, Department of Entomology, and Department of Evolution, Ecology and Behavior, University of Illinois, Urbana, Illinois, United States of America; 6 Department of Biology, University of Utah, Salt Lake City, Utah, United States of America; 7 Department of Entomology, California Academy of Sciences, San Francisco, California, United States of America; 8 National Laboratory for Ecological Analysis and Synthesis (LANASE), ENES, UNAM, Morelia, Mexico; 9 Biology Centre of Czech Academy of Sciences, Ceske Budejovice, Czech Republic; 10 Ecology and Evolution Unit, Okinawa Institute of Science and Technology Graduate University, Onna, Japan; 11 Evolutionary Genomics Research group, Australian National University, Canberra, Australia; Centre National de la Recherche Scientifique, FRANCE

## Abstract

Evolutionary innovations underlie the rise of diversity and complexity—the 2 long-term trends in the history of life. How does natural selection redesign multiple interacting parts to achieve a new emergent function? We investigated the evolution of a biomechanical innovation, the latch-spring mechanism of trap-jaw ants, to address 2 outstanding evolutionary problems: how form and function change in a system during the evolution of new complex traits, and whether such innovations and the diversity they beget are repeatable in time and space. Using a new phylogenetic reconstruction of 470 species, and X-ray microtomography and high-speed videography of representative taxa, we found the trap-jaw mechanism evolved independently 7 to 10 times in a single ant genus (*Strumigenys*), resulting in the repeated evolution of diverse forms on different continents. The trap mechanism facilitates a 6 to 7 order of magnitude greater mandible acceleration relative to simpler ancestors, currently the fastest recorded acceleration of a resettable animal movement. We found that most morphological diversification occurred after evolution of latch-spring mechanisms, which evolved via minor realignments of mouthpart structures. This finding, whereby incremental changes in form lead to a change of function, followed by large morphological reorganization around the new function, provides a model for understanding the evolution of complex biomechanical traits, as well as insights into why such innovations often happen repeatedly.

## Abstract 2

The mousetrap-like mandibles of trap-jaw ants are a functional innovation capable of record-breaking acceleration. This study uses phylogenomics, X-ray microtomography and high-speed videography to reveal that they evolved multiple times around the world through a pathway of incremental steps.

## Main text

Evolutionary change is marked by occasional breakthroughs in organismal design, often involving the reorganization of parts into new functional systems [1,2]. These innovations allow organisms to exploit new ecological niches, change the structure of communities, and impact the organization of biodiversity from local to biogeographic scales [3]. However, understanding how transitions in function evolve when they require changes in multiple interacting parts remains a major challenge. While most agree the evolution of new complex features involves sequences of gradual changes [4–6], these transitional pathways are not yet well understood across systems and different types of traits [7]. For example, investigations into the evolution of biochemical pathways have centered on whether mutational steps preceding the appearance of new functions are adaptive, neutral, or even maladaptive [7,8]. When a trait is both structurally and functionally highly derived from an ancestor, a related question is the extent to which many precursory changes are necessary to “discover” a new function, or whether functional changes occur early and subsequent phenotypic change is driven by selection to explore a new adaptive landscape (i.e., a type of “major” or “key” innovation leading to morphological diversification [9]).

**Fig 1:**
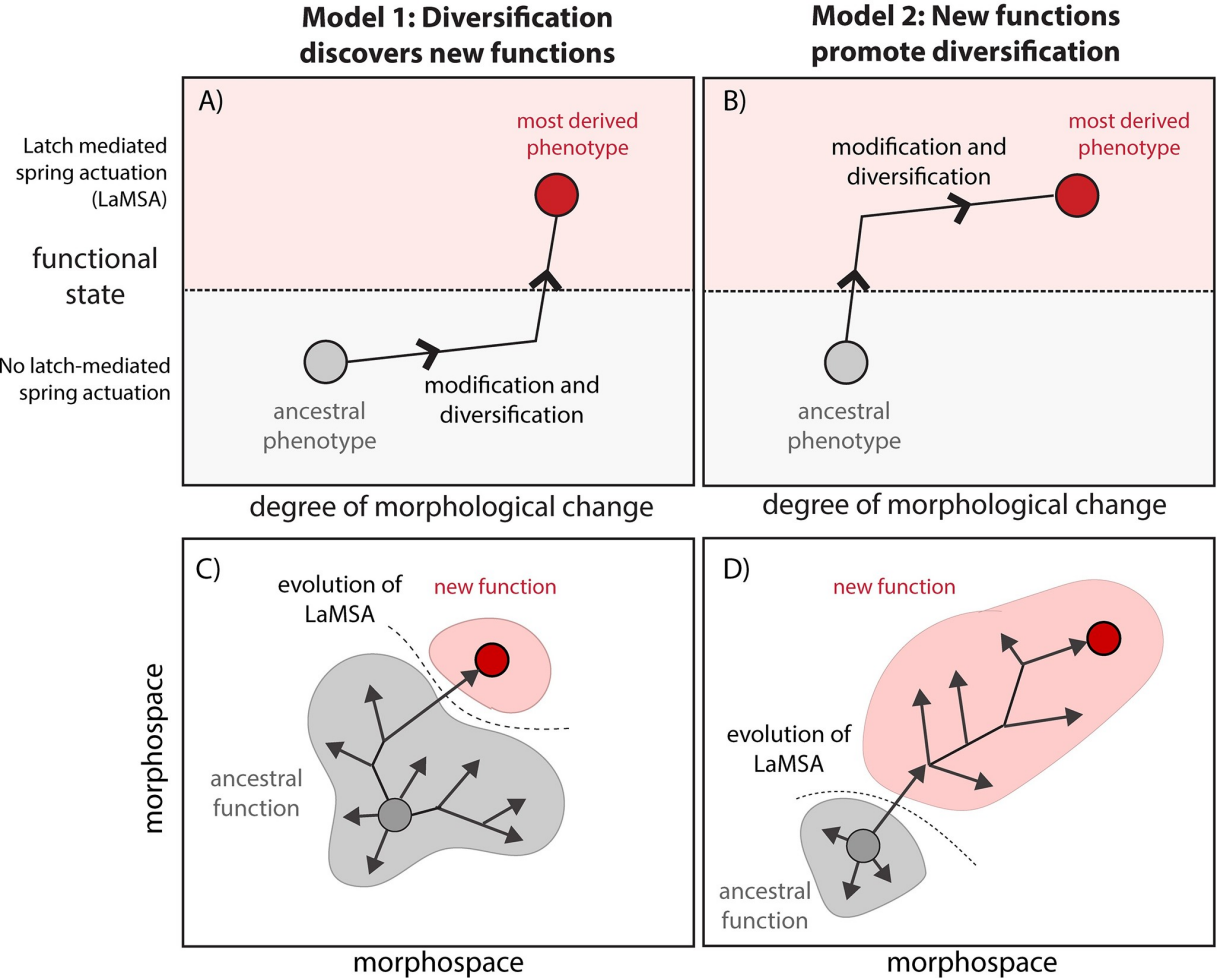
Conceptual diagram showing contrasting hypotheses for a functional and morphological transitions during biomechanical innovation. Biomechanical innovations involve both functional and morphological changes. In one model (A, C), derived morphologies (in this case, modification of the mandible apparatus) diversify first either through neutral variation or adaptation to other functions, and only finally does the new emergent function (in this case, latch-mediated spring actuation, LaMSA) evolves after morphology is highly derived. Alternatively (B, D), the new function evolves through very minor morphological changes, followed by subsequent large diversification in form to explore a new adaptive landscape, reaching new optima for the new function. Our study supports the latter model for trap-jaw mandibles in *Strumigenys* ants.

This question—whether the new function facilitates changes in form or whether the diversification of forms lead to the evolution of new functions—can be asked about biomechanical innovations as well (Fig 1). For example, some of the most extreme, high-performance animal movements involve latch-mediated spring actuation (LaMSA) mechanisms to gradually store and quickly release energy, overcoming the inherent limits to the power output of a motor—often biological muscle [10,11]. Such power amplification mechanisms have become important models for the evolution of biomechanical systems (e.g., [12–14]). The evolution of LaMSA function is often (although not always) associated with large morphological changes relative to ancestors without amplification mechanisms, but did LaMSA evolve before or after major morphological divergences? Unusual morphologies might arise through neutral variation or through selection on ancillary functions that lead to the discovery of a new LaMSA mechanism in an already morphologically derived form (Model 1 in Fig 1). Alternatively, a small morphological change could result in the evolution of LaMSA, which subsequently leads to a large morphological change as selection drives exploration of a new adaptive landscape and the phenotype optimizes around the new function (Model 2 in Fig 1).

Understanding how innovative transitions occur leads to the related question of how likely they are to occur repeatedly. If new, complex phenotypes are readily accessible through pathways of incremental change, or if new functions can evolve first through minor changes to form and then natural selection can then optimize the same set of mechanical tradeoffs, then we may expect these innovations to happen repeatedly. In this scenario, lineages can explore the breadth of an adaptive landscape, leading to repeated discovery of a common set of adaptive peaks. Alternatively, any given breakthrough innovation may be so improbable that it is unlikely to be repeated. In such a scenario, evolution is driven by progressive steps that lead to new areas of phenotypic space, but these are fortuitous and idiosyncratic, such that lineages in different geographic areas are likely to take divergent paths and reach different outcomes.

We examined the evolution of an iconic biomechanical adaptation, the mousetrap-like mandibles of “trap-jaw” ants, to address these general questions about the nature and repeatability of biomechanical innovations. Trap-jaw ants have a latch-spring (LaMSA) mechanism that stores and quickly releases energy, resulting in mandible closure at tremendous speeds. Trap-jaw mandibles have evolved at least 4 times in distantly related ant lineages with different design elements to achieve power amplification in each case [10,15–20], an example of “many-to-one” mapping of form to function [21]. The repeated evolution of similar LaMSA mechanisms in distant lineages is itself fascinating, yet in no case do we understand how these functional breakthroughs occurred from ancestors lacking a trap mechanism.

One globally distributed, hyperdiverse (950+sp.) clade of ants (genus *Strumigenys*) contains a broad array of mandible types, including those both with and without trap-jaw mechanisms, which makes this genus an ideal system for examining the evolution of such mechanisms. *Strumigenys* are leaf-litter predators that primarily feed on springtails (Collembola: Entomobryidae) [22,23], a highly abundant prey that themselves have a spring-loaded escape mechanism. *Strumigenys* mandible type correlates with feeding behavior and microhabitat preference [23], with shorter non-trap-jaw mandibles used for gripping and stinging prey (sometimes using chemical lures [24]), and trap-jaw mandibles used for more active hunting, striking, and stunning prey [23] (S1 Fig). Due to their elusiveness, relatively few predators are known to specialize on collembola (e.g., some salamanders [25], spiders [26], beetles [27], and ants [23]), and arthropods that do often evolve prey capture mechanisms to facilitate capturing them and other fast-moving litter insects. These include adhesive appendages [28], antennal setal traps ([27,29], and the trap-jaw mandibles described here. Collembola have been around since the Paleozoic (Devonian approximately 400 mya), and specialized predatory beetles (Staphylinidae) have records from the Cretaceous (approximately 100 mya), a time when diverse collembola taxa are also abundant in amber deposits [27]. *Strumigenys* diversified more recently (approximately 37 mya, [30]) apparently to exploit this existing and highly productive predatory niche.

All trap-jaw ants close their mandibles using the same mandible adductor muscles (i.e., the motor), pushing against some structure providing resistance (i.e., the latch) until this resistance is removed or some threshold value is exceeded. The *Strumigenys* trap-jaw mechanism is particularly complex; it includes a latch component morphologically uncoupled from the mandible and controlled via contraction of the labrum muscles, an entirely separate muscle group [16]. However, the trap-jaw mechanism has only been studied for 1 species of *Strumigenys* [16], and the diversity of mandible designs has not been widely surveyed. In the species that has been previously studied [16], the morphology of the entire mandible apparatus—including mandibles, muscles, nervous (sensory/trigger neuron), and latch elements—is highly divergent relative to known species that lack a trap-mechanism [16], raising the question of whether these differences evolved before or after the evolution of a latching function. A large-scale comparative study can both illuminate the diversity of mandible designs and provide insights to their evolutionary relationships.

We reconstruct a new phylogeny of *Strumigenys* including 450 species, and survey mandible form and function across this global radiation using physical examination, X-ray microtomography, 3D modeling, and high-speed videography. First, we use these data to ask whether the range of extant forms represent a plausible pathway of intermediates between the ancestral gripping-type mandibles and most derived long-mandibled trap-jaw forms, indicating gradualism as a mechanism of evolving a new complex trait. Second, we ask if the range of phenotypic variation indicates which occurred first: the diversification of morphology (reshaping of the mandible system), or basic functional design of that system (presence or absence of a LaMSA mechanism). In the former model (Fig 1A–1C), we would expect that the mandible apparatus diversifies morphologically before evolving the LaMSA mechanism that changes basic functioning of the system. This is plausible because ant mandibles are highly diverse, and while many species have a stereotypical triangular form, mandible morphology varies considerably, and unusual shapes (such as the long, linear mandibles of trap-jaw *Strumigenys*) do evolve without trap-jaw mechanisms. It is possible that many such changes from the typical mandible system design, at first incidental, are necessary for ultimately accessing the new function. Alternatively (Fig 1B and 1D), LaMSA could evolve through very minor adjustments to the gripping mandibles, but this change opens up a new adaptive landscape for subsequent diversification, resulting in a broader array of mandible apparatus designs across lineages that all have the LaMSA mechanism. We distinguish these 2 alternatives by broadly surveying morphological diversity across the group in a phylogenetic context, and asking whether most morphological diversity of the mandible system is found in forms that have the LaMSA mechanisms, or whether only highly derived morphologies are associated the LaMSA function. Finally, by analyzing the global radiation of this ant group, we assess the extent to which the evolution of mandible diversity was driven by singular evolutionary events versus the repeated evolution of parallel adaptive forms, and whether there is any evidence of biogeographic replication of evolution.

## Results and discussion

### Evolution of mandible systems in *Strumigenys*

**Fig 2:**
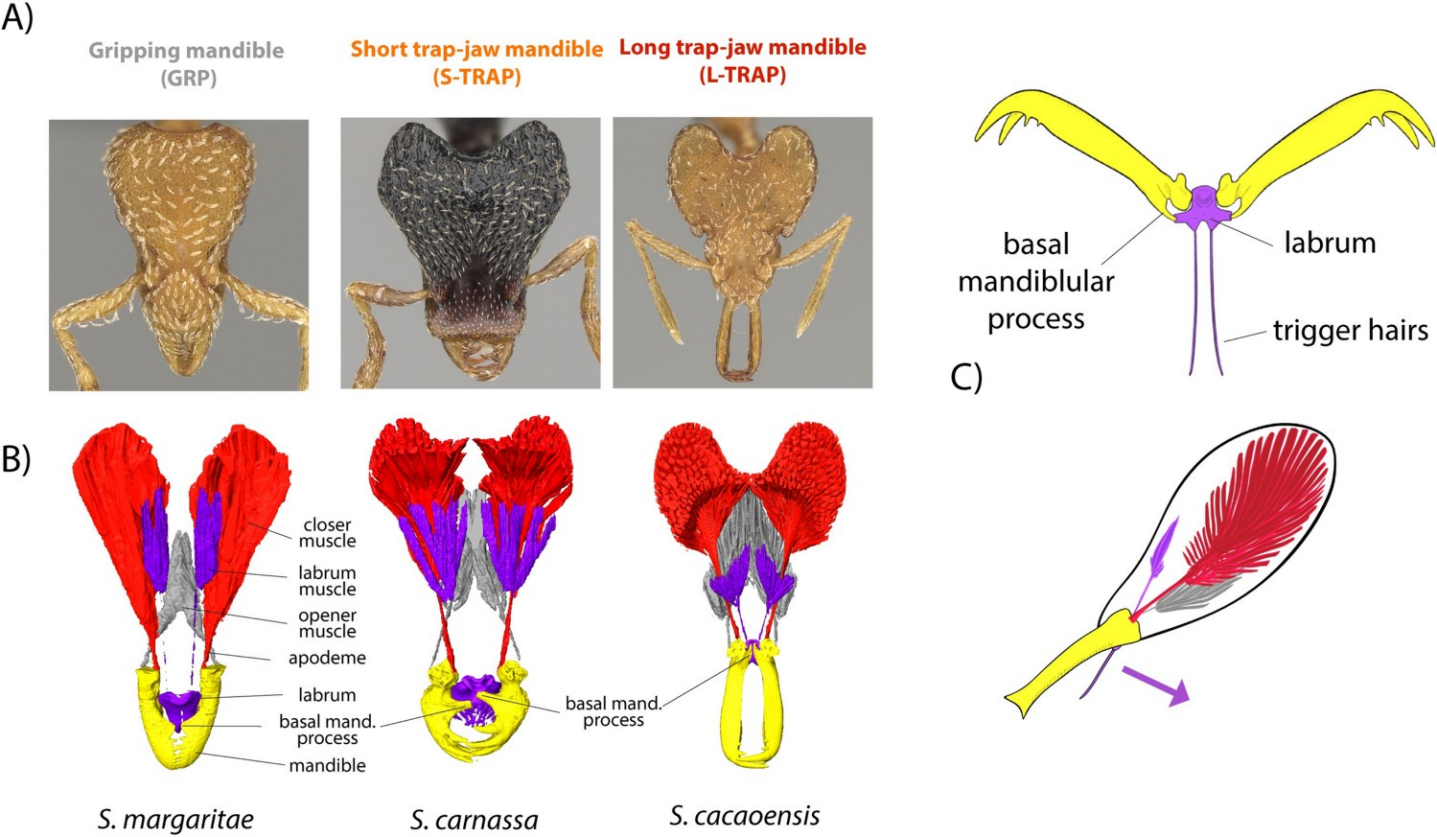
Mandible diversity and trap-mechanism of *Strumigenys* ants. *Strumigenys* is a pantropical, hyperdiverse genus of leaf litter predators. (A) Many species have normal mandibles which they use to grip and hold their prey. Some species have power-amplified “trap-jaw” mandibles which they use to strike and stun prey, which comes in short-mandibled (S-TRAP) and long-mandibled (L-TRAP) forms. (B) The trap-jaw mechanism (seen here in a segmented X-ray micro-CT image) involves the modification of the labrum and basal mandibular process into a latch which locks the mandibles in an open position, with labrum muscle triggering release and mandible closure. The trap mechanism is associated with the reorganization of the muscle fibers, a transition from fast to slow muscle fiber orientation, and other changes to the head design (described further in S1 Text). (C) With trap-jaw mandibles in the locked position, the labrum and basal mandibular processes form a latch mechanism, controlled by rotation of the labrum in the sagittal plane.

**Fig 3:**
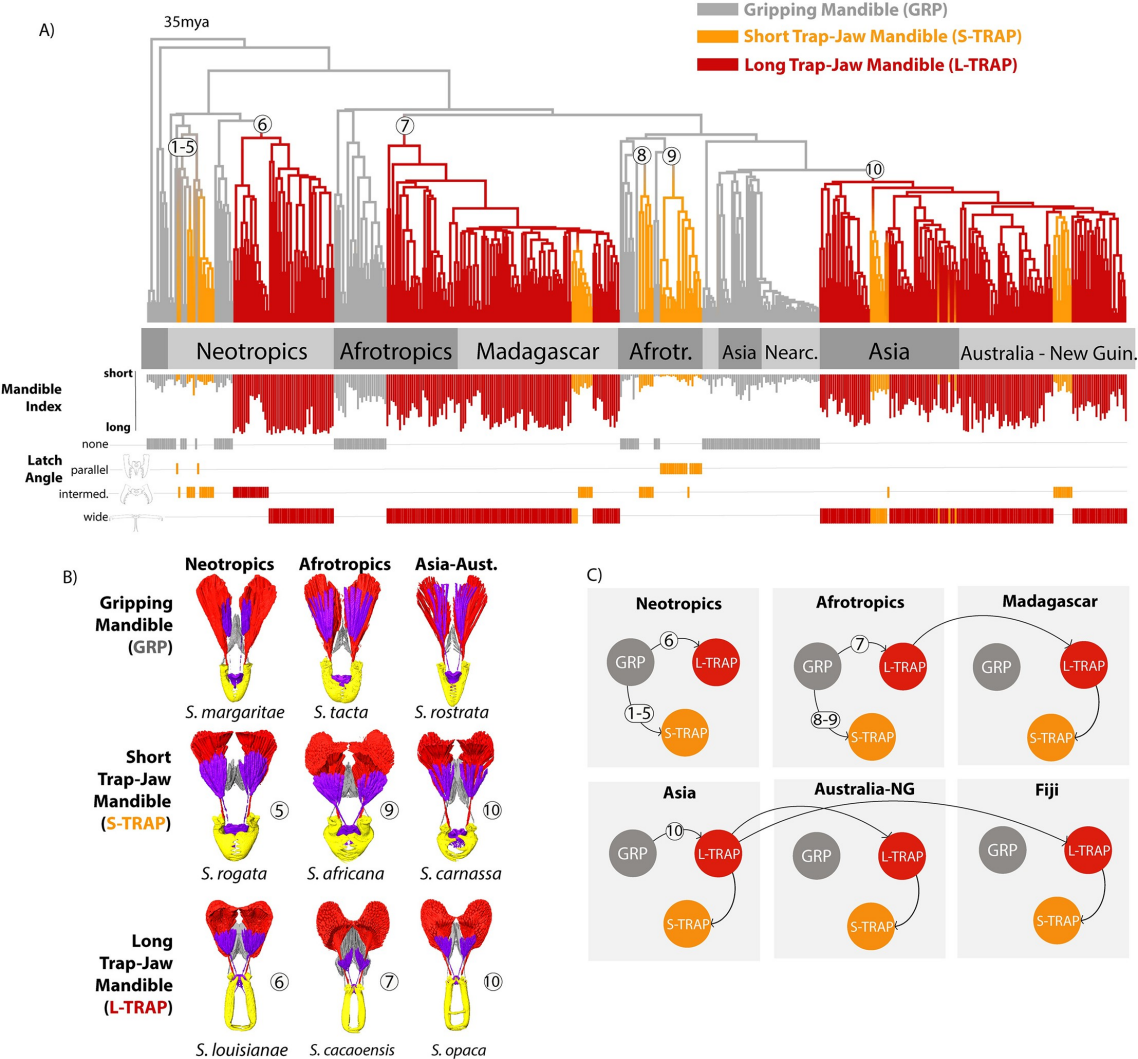
Mandible power-amplification mechanisms and associated ecomorphological diversity have evolved repeatedly from gripping ancestors in *Strumigenys*. (A) The dated maximum likelihood phylogeny of 470 *Strumigenys* species, annotated with estimated locations of the evolutionary transitions to power amplification (white circles, with numbers indexing the different events, not the number of transitions on each branch), and ancestral state estimations of 3 mandible morphs inferred through stochastic character mapping. Tips are annotated with mandible index (mandible length relative to head length) and latch angle (how wide the mandibles open when they reach the latch position). See S3–S5 Figs for trees with detailed annotations and S2, S3 Figs for ancestral state reconstructions with different model assumptions. (B) Comparison of mandible morphs across the world based on segmented X-ray micro-CT imagery, annotated by the LaMSA evolution event (white circle, corresponding to tree). (C) Pathways to ecomorphological diversity through evolution and dispersal among regions inferred with ancestral state estimation (also see S8 Fig).

We examined the breadth of mandible diversity in *Strumigenys* using physical examination, linear morphometrics, X-ray microtomography, and 3D geometric morphometrics, and placed this diversity in an evolutionary context by reconstructing the group’s global phylogeny. Traditionally, *Strumigenys* species have been sorted broadly into 2 forms, those with short triangular mandibles (GRP, Fig 2) used for gripping prey and lacking power amplification, and those with a trap-jaw mechanism associated with long, linear power-amplified mandibles (L-TRAP) used as a high-speed weapon for striking prey. For this study, we examined hundreds of *Strumigenys* species from around the world. Unexpectedly, we found that many of the short-mandibled forms also have a trap-jaw mechanism (henceforth, S-TRAP, Fig 2), based on our examination of whether the labrum and mandible can form a latch which locks the mandible in an open position. Trap-jaw mandibles in *Strumigenys* all have the same basic design in terms of the functional roles of different parts, even as the trap mechanism has evolved repeatedly in the genus. Notably, when present the latch mechanism is always formed through an interaction between a modified labrum and enlarged basal processes of the mandible. Transition to a trap mechanism is associated with similar redesigns of head musculature (see Figs 3 and 5, S3 and S4 Movies, and extended discussion in S1 Text), including reorganization of the muscle fiber structure from speed-optimized to force-optimized. It is not obvious that the *Strumigenys* trap-jaws would all follow the same design, as distantly related ant lineages (e.g., Odontomachini, *Myrmoteras*) have evolved fundamentally different trap-jaw designs that achieve similar functional outcomes [15]. Within this general design, continuous variation exists for nearly all of the traits associated with the trap mechanism, including mandible length and the angle at which mandibles are held in the latched position. In trap-jaw species, mandibles vary from no longer than GRP forms to extremely elongate, and latch angles range from subparallel to 270°.

To investigate the evolutionary relationships between these forms, we reconstructed a phylogeny of 470 *Strumigenys* species from around the globe, including representatives of all known mandible morphologies (Fig 3, S1–S8 Figs). We then used ancestral character estimation to map the evolutionary history of LaMSA and the 3 mandible types (GRP, L-TRAP, S-TRAP, Fig 2) on the phylogeny. Our analysis revealed that while simple, gripping mandibles (GRP) are ancestral, the trap-jaw mechanism has evolved 7 to 10 times independently in different regions, with geography—but not mandible type—being the dominant signal on the phylogeny (Fig 3, S2–S9 Figs, and see S2 Text for further detail of the number of trap-jaw evolutions). The L-TRAP evolved in the Neotropics, Afrotropics, and Asia, and subsequently colonized Madagascar and Australasia. The S-TRAP evolved several times directly from GRP in the Neotropics and Afrotropics but evolved through secondary shortening from the long-mandibled form in Madagascar, Asia, Australasia, and even the small archipelago of Fiji.

This complicated phylogenetic and biogeographic history has led to a common outcome in terms of diversity: All 3 forms are present in each biogeographic region due to different combinations of convergent evolution and dispersal (Fig 3C). Previous work has argued that these forms are “ecomorphs,” in that they are morphologies associated with particular ecological niches [23]. Mandible length in *Strumigenys* is associated with microhabitat, hunting behavior, and diet, with short-mandibled forms more subterranean passive hunters and long-mandibled forms more active hunters on open surfaces [24,31–35]. Thus, the repeated assembly of this ecomorphological diversity globally is reminiscent of the deterministic community assembly observed in some island systems [36,37].

### Convergent evolution of ultrafast performance

**Fig 4:**
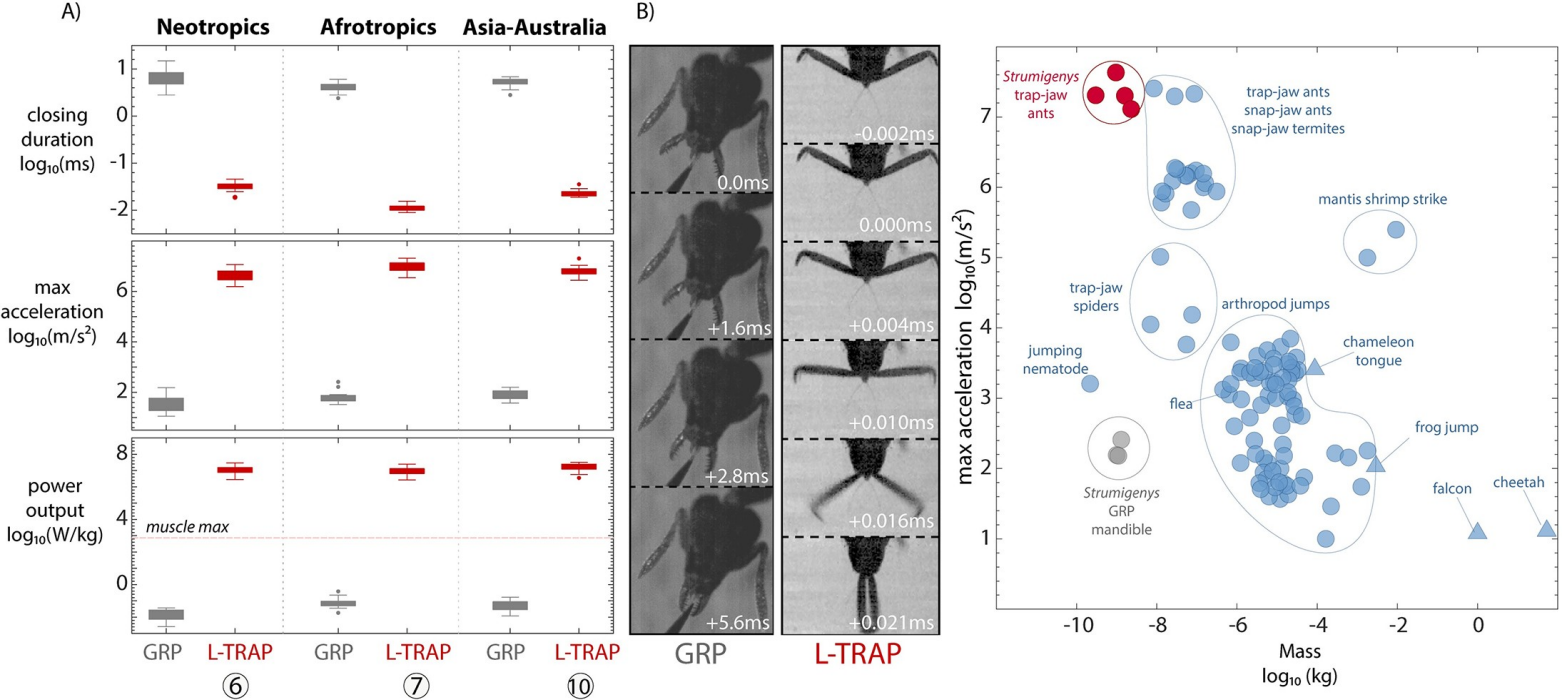
Parallel evolution of enhanced performance associated with the trap-mechanism. (A) Mandible closing duration, maximum linear acceleration, and power output measured with high-speed videography (boxes are middle 50% of data, whiskers are +/−2.7 s, points are outliers). The horizontal dashed line reflects the maximum power output known from biological muscle (730 W/kg) without an amplification mechanism. Each pair represents an independent evolution of power amplification and a related gripping lineage from the same clade. The measured species are, from left to right, *S*. *depressiceps* (*n* = 18), *S*. *elongata* (*n* = 17), *S*. *simoni* (*n* = 14), *S*. *rogeri* (*n* = 12), *S*. *ohioensis* (*n* = 15), *S*. *emmae* (*n* = 20). (B) Example photos from the performance measurement experiments (GRP: *S*. *ohioensis*, L-TRAP: *S*. *elongata*). (C) Power-amplified *Strumigenys* mandibles exhibit the fastest acceleration of any resettable biological movement measured thus far, here L-TRAP species are compared with other invertebrates (circles) and vertebrates (triangles) compiled by Ilton and colleagues [10] and several other studies. The values for mass reflect the moving part or for jumping organisms, the whole body. The data underlying this Figure may be found at [https://doi.org/10.5061/dryad.d7wm37q0t] [41].

Our measurements show that the evolution of the trap-jaw not only leads to extraordinary mandible performance, but increased performance also converges on similar values of acceleration and power output among lineages. We measured the kinematics of several trap-jaw forms derived from independent transitions to power-amplification and found consistent changes in performance, including a 6 to 7 order of magnitude increase in mandible acceleration and a 3 to 4 order of magnitude decrease in strike duration (Fig 4, S2 Movie). The estimated power output for trap-jaw species was also several orders of magnitude higher than GRP species as well as the recorded maximum for biological muscle contraction, supporting our interpretation of the presence/absence of power amplification among the morphs. We compared the performance of *Strumigenys* mandibles to other known ultrafast biological movements and found that the trap-jaw mechanism facilitates the fastest recorded accelerations of any resettable animal movement (Fig 4B). The GRP mandible, while many orders of magnitude slower than the trap-jaw mechanism, is now the fastest ant mandible without a LaMSA mechanism relative to the fastest previously measured species (non-trap-jaw *Strumigenys* have an angular velocity 5 to 10 times faster than *Camponotus floridanus* [38] and have a closing duration half as long as *Harpagnathos saltator* [38]), implying there was selection on mandible speed before the mechanism evolved.

### A pathway of transitional forms supports “function first” evolution of the mandible system

**Fig 5:**
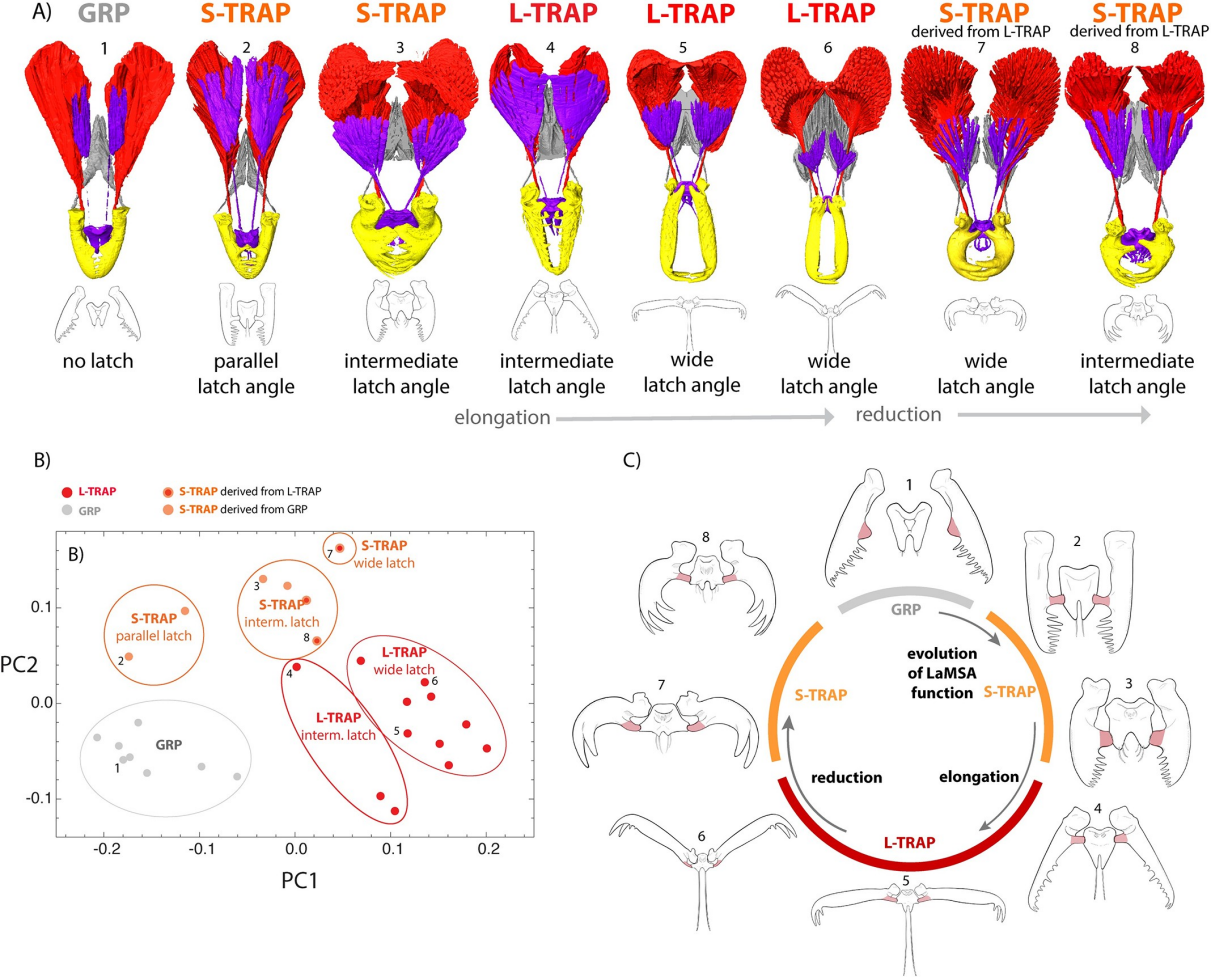
Extant forms represent a continuous pathway between ancestral and most derived mandible types, but this diversity arose after change of function. (A) Across the phylogeny, forms exist which represent nearly every morphological step between the simple gripping mandible (left) to the most derived long-mandibled form (fifth from left), and partial reversion as long-mandibled forms reduced to short-mandibled trap-jaw forms in Asia, Australia, Madagascar, and Fiji. However, most of the morphological diversity arose after a change of function to LaMSA, as seen in (B) the different forms placed in morphospace (PCA) by 3D geometric morphometrics (see S9 Fig for landmark system). (C) A minor change—articulation of the basal processes (pink) with the labrum—changes the function of the labrum to a latch, where the labrum muscle now controls the latch and can unlatch the mandibles to release elastic strain energy stored in the mandible muscle. After this initial functional change, the mandible systems reshape and explore new regions of morphospace, both evolving short and long-mandibled trap-jaws (shown here in latched position). Please also see animated versions of this Figure (S3 and S4 Movies). The data underlying this Figure may be found at [https://doi.org/10.5061/dryad.d7wm37q0t] [41].

The breadth of mandible forms, combined with the reconstructed phylogeny, provides insights into how this innovation may have evolved. First, these forms collectively trace an incremental anatomical pathway between gripping mandibles and the most derived trap-jaw mandibles (Fig 5, S3 Movie), supporting a view of gradualism in the evolution of complex traits. However, the latch-spring-actuation mechanism is present even in forms with very little morphological difference from the ancestral type, and most of the morphological diversity is found among different forms with a latch mechanism. Some S-TRAP forms have mandibles that latch in a nearly parallel position and differ from GRP forms so subtly that they are challenging to distinguish without relaxing and manipulating the labrum and mandibles into a latched position. These S-TRAP forms provide clues to the initial transition from gripping to trap-jaw mechanism. In contrast to typical triangular ant mandibles, all *Strumigenys* have a flattened basal process at the angle between the basal and masticatory margins of the mandible (bmp, S11 Fig). In gripping species, the function of this process is unknown, but this structure is well positioned to be co-opted to interact with the labrum and form a latch. We observed the labrum itself used by gripping species as a sensor between the mandibles. Among these ants, the labrum is pulled down and out of the way during mandible closure (Fig 5, S2 and S3 Movies). Once the basal process starts to contact the labrum during mandible closure, it creates the potential for elastic energy storage, with the muscle that moves the labrum now serving as a mechanism to control latch release and mandible closure. In this way, a slight realignment of an existing structure led to a new function. Following the initial functional change, optimization for the new function can drive toward a new adaptive peak, leading to the reorganization of head design described above (Fig 5).

These results for trap-jaw ants echo previous comparative studies of the evolution of high-performance systems. In a broad analysis of the evolution of snapping shrimp claws, Kaji and colleagues [39] found that subtle changes in the claw joint morphology led to large changes in the function of the claw, ultimately leading down an evolutionary path toward remarkably ultrafast motions capable of cavitation, and this innovation happened in parallel in different lineages. As with the slight changes to the mandible morphology seen in our study, changes to the claw joint were so subtle that the functional implications were overlooked, and the change of function preceded a remodeling and diversification of the claw joint during a large evolutionary radiation. Similarly, the spring-powered tongues of some salamanders evolved in parallel across different lineages through small morphological changes that had large functional effects [12].

## Conclusions

Our analysis of the evolution and diversification of *Strumigenys* mandible systems provides insights into the evolution of complex biomechanical traits in general, and LaMSA in particular. We find *Strumigenys* has more diversity in mandible type than previously documented, including many intermediate phenotypes that provide clues to the transitional paths between the ancestral, triangular, gripping mandible and the most derived, long-mandibled trap-jaw. According to our analysis, this diversity is found mostly among lineages that have already evolved LaMSA. Furthermore, the transitional forms show that extremely modest morphological changes can result in the evolution of a latch-spring-actuation mechanism. This supports a view that function evolved first, followed by diversification and exploration of a new adaptive landscape associated with the new function, and eventually developing into the animal kingdom’s fastest-accelerating resettable part. We propose that this phenotypic adjacency explains why the mechanism has evolved so many times independently around the world. If minor morphological changes can lead to new functions among the parts, and subsequent pathways of continuous, incremental changes facilitate exploration of an adaptive landscape associated with the new function, one would predict the repeated discovery of different ecomorphological forms and the deterministic evolution of diversity across space and time. When our results are considered together with other systems [12,39], a picture emerges for how morphologically and functionally derived high-performance systems evolve—first through subtle morphological changes with large functional effects, followed by morphological remodeling around the new function. Collectively, these findings move us toward a more general theory of how form and function change during the evolution and diversification of biomechanical systems.

## Materials and methods

### Taxon selection

*Strumigenys* is a pantropical, hyperdiverse genus (843 described species, the third most speciose ant genus [40]) divided into 116 morphologically distinct species groups [22]. We sampled a taxon set covering most of the morphological and geographical diversity within the genus, using material from personal and museum collections. Bolton [22] performed a comprehensive global revision of the genus and created morphologically based species groups, giving us a benchmark for sampling global diversity. After removing specimens that had poor quality extractions or did not yield enough sequence data, the final taxon set used to reconstruct the phylogeny included 885 specimens from 470 species (360 described and 110 undescribed species, listed in Dataset 1 in a Dryad repository, https://doi.org/10.5061/dryad.d7wm37q0t [41]). This set covers 90% of all Bolton species groups (104/116) and included representatives from all main geographic regions. A number of undescribed species included in our taxon set did not conform to descriptions of any of Bolton’s species groups and appear to belong to 8 additional species groups. Of these, 4 species could not be determined for trap-jaw status due to damaged material, thus the phylogeny used for the ancestral state analyses included 466 species. We also examined further hundreds of species that were not sequenced; these were used to inform our understanding of morphological variations across the group but were not used in the formal analyses.

### Determining presence-absence of a trap mechanism

We inspected the labrum and basal mandibular process to determine whether each species had trap-jaw mandibles (TRAP) or gripping mandibles (GRP). To qualify as TRAP, the species must have an identifiable latching mechanism. All TRAP *Strumigenys* have a basal mandibular process that inserts into a lateral indentation on the labrum, forming a latch when the mandibles are fully open. In many species (both TRAP and GRP), the presence or absence of a complementary mandibular process and lateral labral pocket could be seen without manipulation of the specimen. For any species not showing clear visual confirmation of TRAP or GRP morphologies, we manipulated the labrum and mandible to assess whether they could be maneuvered into a latching position. To facilitate this examination, we softened specimens in a 50% water/ethanol mix for 0.5 to 2 hours. For TRAP species, we confirmed the mandibular process would lock mandibles open against the lateral labral pocket. In GRP species, we confirmed that the labrum and basal mandibular process did not come into contact and that other potential locking mechanisms were not present. We visually inspected every species of our taxon set and examined the opening position of mandibles and labrum of one or more species from each main clade and morphotype on the tree (*n* = 132 species examined through manipulation).

### Measurement of mandible index and mandible opening angle

For described species, linear morphometrics were obtained from original descriptions, from Bolton’s revision [22], or from new measurements taken for undescribed species. We checked the data for outliers and remeasured any specimens that appeared questionable. New measurements were made digitally using the Leica LAS X Measurement software using a Leica M 165C stereoscope equipped with a Leica IC90 E Camera. When original descriptions gave a range of measurements not associated with individual specimens, we used the median value of the range, or if data for multiple specimens were available for a species, we took the mean. In this study, we used Mandible Index, the ratio of Mandible Length (measured from the tip to the frontal margin of the clypeus) to Head Length (the frontal margin of the clypeus to the posterior margin of the head in frontal view). We also measured the locking angle, the angle between 2 joining lines drawn along each mandible from the base of the apical tooth through the base of the basal mandibular process (S11 Fig). The measurement was taken while the mandibles were held or positioned so that the basal mandibular process rested in the complementary labral pocket.

### Micro-CT scanning, segmentation, and 3D geometric morphometrics

We used X-ray microtomography to scan 45 selected representatives of different species groups and examine the design of the mandibles (Dataset 2, in the Dryad repository https://doi.org/10.5061/dryad.d7wm37q0t [41]). Scans were performed either with a Zeiss Xradia XRM510 (Okinawa Institute of Science and Technology) or a Zeiss Xradia XRM400 (University of Illinois). Specimens were either iodine stained in alcohol and scanned, or fixed in alcoholic Bouin solution, iodine stained in ethanol, and critical point dried prior to scanning. If necessary, unstained, point-mounted specimens were used. The musculoskeletal system is typically still intact in dried specimens, although some shrinkage between fibers can change the appearance. We then examined the scans visually to assess the mandible mechanism. For 27 species represented of the different forms/clades, we segmented the mandible system (including mandible, labrum, labrum muscle, adductor, abductor, and apodeme) to produce visualizations using Amira version 6.2 and used geometric morphometrics to quantify the 3D morphology of the mandible system. Our purpose was to use landmarks to capture the overall variation in shape dimensions and arrangement of the system components, not represent strict homologies which can be difficult to apply to muscle fibers. We first exported surfaces of all segmented mandible systems as.ply files using *extract_surface* function in Amira. Those surfaces were later imported into Meshlab version 2016.12 for post-processing to generate hollow surface models. We then used *Checkpoint* software (Stratovan, Davis, California) to place landmarks on the mandible system of each specimen. In total, a dataset of 12 landmarks were placed on 27 specimens (see S10 Fig for landmark definitions). Geometric morphometric analyses were conducted using the R package *geomorph* [42]. We first apply a Generalized Procrustes Analysis of our 3D-landmark dataset to align and rescale the landmark system. We then visualized variations in shape space using a Principal Component Analysis (PCA) using the function *PlotTangentSpace*.

### DNA sequencing and phylogeny reconstruction

We used RAD-seq [43] to generate molecular data for phylogeny reconstruction, as we wanted a high-throughput protocol that is robust to high DNA degradation as is typical in museum specimens and older field collections. We extracted DNA following the protocol described by Tin and colleagues [44], digested the DNA with the restriction enzyme EcoRI, and prepared libraries following Tin and colleagues [45] using the Biomek FXP Laboratory Automation Workstation (Beckman Coulter, Brea, California). Libraries were sequenced single-end with 55 bp read length on an Illumina HiSeq 2500 platform in the DNA sequencing section (SQC) at the Okinawa Institute of Science and Technology Graduate University. Samples were demultiplexed, filtered by quality, and trimmed to 42 bp using Trimmomatic [46]. We used *pyrad* v.3.0.66 [47] for de novo assembly of RAD loci of 1,189 specimens (parameters: mindepth: 6, NQual: 4, Wclust: 0.88, MaxSH: 3, MaxH: 8, otherwise default settings). After assembly, we reduced this set to 885 specimens by filtering out specimens with low data coverage and trimmed each locus to 30 bp by shortening the tail end, where mapping errors are more likely.

While RAD sequencing generally recovers many loci, it often results in large amounts of missing data across specimens. However, previous studies have shown that including loci with low coverage across taxa is beneficial for phylogeny reconstruction, because even loci present for small numbers of species are informative about internal nodes in the tree [48]. Our experience supports this conclusion. The single-digest cutter we used (EcoRI) is less sensitive to mutation disruption, which allows for more phylogenetic depth but increases the number of loci, which in turn increases missing data for a given sequencing effort. Lower sequencing coverage allows for more cost-effective sequencing of many specimens, necessary to sequence large numbers of species. Thus, we included as many loci in the analyses as was computationally feasible, even though that raised the proportion of missing data. Although the assembly recovered 2,296,160 loci (approximately 7 million bp) present in at least 4 individuals, we filtered out loci occurring with low frequencies across specimens, reducing this number to 180,841 loci, with a total alignment of 5,380,522 bp in the alignment (mean 220,041 bp/present per specimen; 343,586 bp present per species). We performed Maximum Likelihood (ML) phylogenetic reconstruction on this dataset using different subsets of the data. First, we made a more inclusive set including specimens with relatively low amounts of data. Second, we used a different approach by reconstructing a “backbone” phylogeny using only species with higher data coverage (132 species). We chose a reduced subset of species that had a high percentage of data and represent different clades in preliminary analysis and found consensus sequence across specimens of each species. We performed ML searches on both alignments using ExaML v3.0.1.17 with the PSR substitution model, with 100 bootstraps, and compared the resulting trees. As the topology was consistent between the different analyses, we used the larger tree for comparative analyses in the study. To date the full ML topology, we pruned the alignment to 1 specimen per species, reduced to a smaller alignment by dropping low-coverage loci, and ran a Bayesian dating analysis in BEAST v2.4.8 [49]. We calibrated the *Strumigenys* crown node with a uniform prior (27.2 to 39.2 MY) following the results of a recent study that places *Strumigenys* in context of the Myrmicinae [30]. We also experimented by running the dating analysis with different levels of data inclusion (e.g., either by prioritizing a larger alignment or minimizing missing data) and found the resulting node ages to be insensitive to these variations. We used Partitionfinder v2.1.1 [50] to choose the nucleotide substitution model (GTR+G+X) for the dating analysis. As a cross validation of our RAD-seq approach, we compared the resulting topology with results from a previous study [51] which used an entirely different molecular dataset. That study was focused on a larger scale reconstruction of relationships across all ants but included 19 *Strumigenys* species. To estimate whether high levels of missing data resulted in a loss of overlapping loci in deeper phylogenetic scales (e.g., due to mutation disruption which would give a phylogenetic signal to missing data), we used the method of Eaton and colleagues [48] to find the number of potentially quartet informative loci on each branch of the resulting tree. We found that deeper branches had far more loci represented than shallow branches (S8 Fig), indicating the missing data was mostly due to low coverage sequencing rather than mutation disruption, and that this allows the accumulation of large amounts of data to inform the internal branches of the tree.

Although the structure of the phylogeny overall was stable across different analyses and methodological assumptions, the rooting of the genus was unstable in our analysis, probably due to low data overlap with distant outgroups. Specifically, our analysis could not distinguish between 2 different rootings (notably, whether the *capitata* and *ambatrix* groups are sister to the rest of *Strumigenys* “root 1,” or whether they are sister to the Neotropical clade “root 2,” forming a clade that itself is sister to the rest of *Strumigenys*, S3 and S4 Figs). To ensure our conclusions about mandible evolution are not sensitive to this uncertainty, we dated the full tree in BEAST2 with each rooting and repeated all ancestral state estimation analyses with both topologies.

### Ancestral mandible character and biogeographic state estimation

We estimated the evolutionary history of trap-jaw mandibles on the phylogeny using parametric models of discrete character evolution. Our primary focus was reconstruction of the trap mechanism as a binary, discrete character (TRAP or GRP), representing the main functional division between mandible types. We fit a variety of models for binary trait evolution in order to examine the sensitivity of the results to model assumptions. First, we fit Mk1 (gains and losses equally likely) and Mk2 (gains and losses can differ in rate) models using ML implemented in the R package *geiger* (fitDiscrete function) [52] and compared models with sample size-corrected Akaike Information Criterion (AICc). To reconstruct the ancestral states under each model, we performed 500 stochastic character maps with each model using the R package *phytools* (make.simmap function) [53]. Because variation in trait evolutionary rates across the tree (i.e., heterotachy) can sometimes lead to mistaken inferences when a time-homogeneous model is used [54], we also fit the Mk2 model using a Bayesian Random Local Clock model implemented in BEAST [55] (following King and Yee [54]) and compared results.

As a secondary analysis mainly used to produce a visualization of the evolution of the 3 main ecomorphs (GRP, S-TRAP, and L-TRAP, Fig 3), we fit a 3-state Mk model using the same process above (although we did not fit 3-state heterotachous models). In general, modeling mandible morphology with 3 states did not change the overall pattern of evolution of LaMSA. Results of all ancestral state estimations were visualized using methods for mapping trait evolution on trees [56] using the package *phytools* [53]. We provide further discussion of ancestral state estimation methods and describe the results in S2 Text.

In order to place mandible evolution in a broad biogeographic context, we also estimated ancestral biogeographic states on the tree. As we only sought to characterize biogeographic history on the broadest scale (6 regions, Afrotropics, Asia, Madagascan, Nearctic, Neotropical, and Oceanian) and nearly all species are limited to 1 region, we used stochastic character mapping (using make.simmap in *phytools* [53]) without allowing species to extend to more than 1 area (in other words, we used an equal-rates Mk model without explicitly modeling range transitions) and visualized the probability of each ancestral node being located in each region in the tree.

### Kinematics measurements and cross-taxon comparison

We measured mandible kinematics of both gripping and trap-jaw forms using high-speed videography. Our purpose was 2-fold: to estimate the influence of the trap mechanism on performance and to confirm power-amplification by comparing performance to maximum predicted power output from biological muscle alone (although expected, this has not been directly confirmed before in this genus). We also measured and compared the performance of different species representing different evolutionary transitions to power-amplification. Colonies of live *Strumigenys* of 6 species representing different clades/ecomorphs (L-TRAP: *S*. *elongata*, *S*. *rogeri*, *S*. *emmae*, *S*. *faurei*; GRP: *S*. *simoni*, *S*. *ohioensis*, *S*. *depressiceps*) were collected (see Dataset 3 for full collection info) and exported to the University of Illinois at Urbana-Champaign with the proper permits, which are available upon request. For species with power-amplification, 2 to 5 ants per species were individually mounted to paper points at the posterior-dorsal part of the head using wood glue and positioned under an SA-Z high-speed camera (Photron USA, San Diego, California) that was either attached to an M165 FC microscope (Leica Camera, Wetzlar, Germany) or a Canon 100 mm macro lens so their mandibles were fully visible and in line with the camera. Ants were backlit with LED lights so their silhouettes were visible, then stimulated to strike 2 to 9 times by gently blowing air at their mandibles. Videos were recorded at 480,000 to 900,000 frames per second. For non-power-amplifying species (*S*. *ohioensis* and *S*. *simoni*), 3 to 5 ants per species were restrained under a Phantom V9.1 high-speed camera (Vision Research, Wayne, New Jersey) attached to a SteREO Discovery V20 microscope (Carl Zeiss, Oberkochen, Germany) by holding the thorax or a hind leg with a pair of fine-tipped forceps. One to 8 strikes per individual were initiated by gently poking the ant in the head with a 0.55-mm thick pin until the mandibles opened, and then touching the inner surface of the mandibles or labrum with the pin. Videos were recorded at 2,500 frames per second. Each ant filmed was killed by being placed in a −20°C freezer. Individual mandible and body masses for each ant were later taken using a UMX2 ultra microbalance (Mettler Toledo, Columbus, Ohio). In some cases, both mandibles were weighed together and averaged to calculate mass. In the case of *S*. *emmae*, which had mandibles too small to be detected by the balance even in pairs, mandible mass was estimated by calculating the average mandible density of all other filmed species using mandible volumes from micro-CT scan data (see below). Using this number and the *S*. *emmae* micro-CT scan, we were able to estimate mandible mass for this species. To estimate the mass of the mandible adductor muscle for each species, the mandible adductor muscles of 3 workers of *S*. *ohioensis* were dissected out, weighed in pairs using the ultramicrobalance, and the average weight per muscle was calculated. This value was then used to estimate the weight of mandible adductor muscles in all other species based on muscle volume taken from CT scan data, assuming equal density of muscle for all species. This was done due to the difficulty of dissecting out the muscles from power-amplifying species, which tend to break up into individual fibers when removed from the head capsule because of the way the fibers are attached to the mandible apodeme.

In each high-speed video, the mandibles were tracked using a MATLAB v14b (Mathworks, Natick, Massachusetts) script taken from Spagna and colleagues [57], and then analyzed in R version 3.2.2 [58] using the function *Trapskin* from Larabee and colleagues [20]. Briefly, this function uses the function *pspline* from the package *pspline* to approximate an angular displacement versus time curve. Rotational velocity and rotational acceleration are then calculated using the first and second derivatives of the displacement versus time curve, respectively. Maximum rotational kinetic energy was calculated by modeling the mandibles as a thin rod of uniform density rotating about one end. This value was combined with estimated muscle mass for each species to calculate the maximum power output of the mandible adductor muscle.

To compare the performance of *Strumigenys* mandibles with other ultrafast movements across the animal kingdom, we used data primarily from the compilation of Ilton and colleagues [10]. In addition, we added data from recent studies [20,59–62] and measurements for the 7 *Strumigenys* species (L-TRAP: *S*. *elongata*, *S*. *rogeri*, *S*. *emmae*, *S*. *faurei*; GRP: *S*. *simoni*, *S*. *ohioensis*, *S*. *depressiceps*) from our video experiments. Data from the new kinetics measurements in this study (Dataset 3) and cross-taxon compilation (Dataset 4) are available in the Dryad repository (https://doi.org/10.5061/dryad.d7wm37q0t) [41].

### Behavioral video

To illustrate prey capture behavior in an L-TRAP species, a colony of (*Strumigenys elongata*) was collected at Amazon Conservatory for Tropical Studies (ACTS) research station in Peru in July of 2018 and transferred to small plaster-filled petri dish for observation. Local springtails were collected live from leaf litter taken from the surrounding rainforest using Winkler extractors, transferred to the petri dish, and allowed to roam freely among the ants until a prey capture event occurred. Encounters between springtails and ants were filmed using a Motorola X2 smartphone (30 fps, default camera app) connected to a Leica S6E stereomicroscope with a Gosky universal cell phone adapter mount.

## Supporting information

S1 Movie Prey capture behavior of a representative L-TRAP species, *Strumigenys elongata*. *S*. *elongata* captures a springtail with its power-amplified mandibles and lifts the springtail by raising its head so that the springtail’s jumping escape mechanism is rendered ineffective. The ant subsequently rotates its abdomen forward to sting and incapacitate the springtail with venom. The video was filmed and is presented at 30 fps.
(MOV)

S2 Movie Representative mandible strike of the Nearctic GRP species *Strumigenys ohioensis* and Neotropical L-PAM species *S*. *elongata*, corresponding to the measurements depicted in Fig 3. The *S*. *ohioensis* video was filmed with a Phantom V9.1 high-speed camera at 2,500 fps and played back at 5 fps. The labrum begins to rotate ventrally out of the path of the closing mandibles shortly after the mandibles begin to close, eliminating the possibility of the labrum acting as a latch during this strike. The *S*. *elongata* video was filmed with a Photron SA-Z high-speed camera at 480,000 fps and played back at 30 fps. In the gripping species, the labrum (located at the end of the pin between the mandibles) rotates ventrally prior to the mandibles beginning to close, disengaging from the basal processes of the mandibles enabling them to close.
(MP4)

S3 Movie Animation of transitions between major morphological mandible forms of *Strumigenys* as depicted in Fig 4; see S11 Fig for terminology; darkest shaded region of mandibles seen through dorsal shield of clypeus; (1) *S*. *margaritae*, (2) *S*. *DBB130*, (3) *S*. *rogata*, (4) *S*. *aethegenys*, (5) *S*. *louisianae*, (6) *S*. *cacaoensis*, (7) *S*. *oasis*, and (8) *S*. *carnassa*. The basal mandibular process and labrum changes from no contact in GRP species *S*. *margaritae* (form 1) to contact and locking (forms 2–8). Although the angle of locking remains near parallel in some clades of *Strumigenys* (form 2), the angle increases in independently derived LPAM clades—suggesting intermediate extant forms represent similar evolutionary stepping stones paths of LPAM. We also provide the same animation sequence showing the mandible and labrum sequence in context of broader changes in head shape. We do not suggest all changes happened following this sequence, only that functional intermediates exist between gripping and trap-jaw forms and represent plausible pathways between forms.
(MP4)

S4 Movie Relationship between forms with different musculature between major morphological mandible forms of *Strumigenys*. The mandible opening muscles are in gray, mandible closing muscles in red, labrum muscles in purple, labrum in purple, mandibles in yellow; (1) *S*. *margaritae*, (2) *S*. *aethegenys*, (3) *S*. *louisianae*, and (4) *S*. *cacaoensis*. Muscle fibers change from “speed optimized” linear arrangement that are confined to the lower half of the head in GRP species *S*. *margaritae* morph into more acutely angled “force-optimized” arrangement and expand into the posterior dorsal portion of the head capsule in more derived PAM species.
(MP4)

S1 Text Further background, analysis, and discussion of *Strumigenys* mandible morphology in the context of the evolution of trap-jaw mechanisms. (PDF)

S2 Text Extended discussion of the number of ancestral state estimation and the number of trap-jaw evolutions. (PDF)

S1 Fig Hunting tactics of short and long-mandibled *Strumigenys* ants. *Strumigenys* is a pantropical, hyperdiverse genus of leaf litter predators whose preferred prey are usually (A) springtails—leaf-litter arthropods with a power-amplified spring-like escape mechanism (furculum). Traditionally, *Strumigenys* have been divided into 2 main ecomorphs [23]: (B) short-mandibled forms, that tend to be more cryptobiotic and subterranean feeders that employ a strategy of luring or cautiously approaching prey, then gripping onto and stinging the struggling prey item, and (C) long-mandibled forms that use the trap-jaw mechanism to strike and stun, lift, then sting their prey, and are more active hunters (also see S1 Movie). We also report here that several groups of short-mandibled forms actually have a trap-mechanism. The drawings are by Mayuko Suwabe.
(PDF)

S2 Fig Ancestral state reconstruction of the presence or absence of trap-jaw mandibles (TRAP) under different models of character evolution under rooting 1. Ancestral state probabilities under (A) the time-homogeneous symmetric (Mk1) model, (B) the time-homogeneous asymmetric (Mk2) models are marginal probabilities of node states from 500 stochastic character maps using the maximum likelihood transition matrix. (C) Results of a Bayesian MCMC analysis of the asymmetric (Mk2) model allowing for rate changes on the tree, implemented in BEAST. In each case, ancestral state probabilities (marginal or posterior) were calculated for each node, then branchwise state probabilities were visualized using the contMap function in R. (D) The maximum likelihood (for Mk1 and Mk2) or posterior means (for Mk2-RLC) for each parameter or inferred number of transitions/rate changes.
(PDF)

S3 Fig Ancestral state reconstruction of the presence or absence of trap-jaw mandibles (TRAP) under different models of character evolution under rooting 2. Ancestral state probabilities under (A) the time-homogeneous symmetric (Mk1) model, (B) the time-homogeneous asymmetric (Mk2) models are marginal probabilities of node states from 500 stochastic character maps using the maximum likelihood transition matrix. (C) Results of a Bayesian MCMC analysis of the asymmetic (Mk2) model allowing for rate changes on the tree, implemented in BEAST. In each case, ancestral state probabilities (marginal or posterior) were calculated for each node, then branchwise state probabilities were visualized using the contMap function in R. (D) The maximum likelihood (for Mk1 and Mk2) or posterior means (for Mk2-RLC) for each parameter or inferred number of transitions/rate changes.
(PDF)

S4 Fig Maximum Likelihood backbone phylogeny of the ant genus *Strumigenys* using only species with the highest data coverage to represent individual clades. The tree, including 132 taxa was inferred with ExaML. Node supports reflect bootstrap (left) and booster (right) scores from 100 bootstraps. The tips are annotated with species name, ecomorph (GRP, S-TRAP, L-TRAP), latch angle, and geographic region. The bars on the right reflect the dominant geographic region for each clade, although some lineages within the clade may be in different regions. The red dot shows the placement of the ambatrix and capitata groups sister to the neotropical clade (we call this “root 2” position). The alternative rooting of the *Strumigenys* clade has *ambatrix* and *capitata* sister to the rest of the *Strumigenys* (see S5 Fig) and was inferred in the full analysis, although neither placement is well supported in the 2 analyses.
(PDF)

S5 Fig The maximum likelihood tree for the full dataset pruned to 1 tip per species and dated in BEAST2. Nodes are annotated with common ancestor ages and 95% height ranges from a posterior sample of a Bayesian analysis. The red dot indicates the position of the *ambatrix* and *capitata* groups in “root 1” position. This is the tree used in the analyses, although we also ran analyses using an alternate topology where the *ambatrix* and *capitata* groups (bottom left) are sister to the neotropical clade (”root 2” position, see S3 Fig).
(PDF)

S6 Fig The maximum likelihood tree for the full dataset (885 specimens) inferred with ExaML. Nodes are annotated with bootstrap and booster scores based on 100 bootstraps.
(PDF)

S7 Fig Consistency of RAD-seq and gene-based phylogenies. To check whether our RAD-seq–based phylogeny was consistent with previous analyses, we compared the topology of subtrees of overlapping taxa between the current analysis (i.e., as seen in S4 Fig) and a previous analyses based on different molecular data (tree from a family wide diversification analysis in Economo and colleagues, most of the data from Ward and colleagues which also inferred a nearly identical topology). In some cases, where the same species was not sequenced, a closely related species from the same species group was substituted for the comparison (*S*. *atopogenys* for *S*. *ocypete*, *S*. *sistrura* for *S*. *olsoni*, *S*. *simoni* for *S*. *ludovici*, *S*. *hubbewatyorum* for *S*. *nitens*), these are denoted with parentheses. Numbers indicate bootstrap support for nodes that disagreed between the 2 trees. Note the 11-gene phylogeny recovered the *ambatrix* group in the “root 1” position, sister to the rest of *Strumigenys* (although *capitata* group was not included).
(PDF)

S8 Fig The number of potentially quartet-informative loci at each branch. In RAD-seq phylogenomics, missing data at tips can either be structured by clade (usually due to mutation-disruption) or randomly across the phylogeny (usually due to low sequencing coverage). Eaton and colleagues showed that while the former is a problem for phylogenetics, because of lack of overlapping data among distant clades to inform in deeper relationships, but the latter is less of an issue. Indeed, including more data present in few individuals is beneficial because it can inform deeper nodes. As deeper branches can be informed by more tips, the number of loci potentially expands dramatically because they have more chances to be recovered in descendents of each branch. Here, we plot the number of potentially quartet informative 30 bp loci for each internal branch (positioned on the node to the right of the branch) of the tree depicted in S4 Fig to be potentially quartet-informative, a locus must be present in 1 tip in among the descendents of each of the 4 branches originating from a focal branch (see Fig 1 in Eaton and colleagues). The accumulation of many informative loci for deeper nodes shows that the pattern of missing data is not highly phylogenetically structured and is thus informative for tree inference on this phylogenetic scale.
(PDF)

S9 Fig Biogeographic history of *Strumigenys*. Probabilities of ancestral states in each broad geographic region calculated with stochastic character mapping under a Maximum Likelihood model. The deepest nodes are uncertain, but the main clades within the genus are highly geographically structured.
(PDF)

S10 Fig 3D morphospace of the mandible system and landmark positions. The inferred PCA morphospace plot for *Strumigenys* (seen in Fig 5) annotated with species names. Example forms with landmarks placed are below. The landmarks were placed as such: L1–L2, the most posterior points of the closing muscles where they attach to the posterior margin of the head; L3–L4, points on the closing muscles where they attach to the middle point of the posterior margin of the head. L5–L6, the most ventral points of the closing muscles where they attach to the ventral side of the head; L7–L8, the points on the apodeme where the closing muscles start; L9–L10, the most anterior points of closer apodemes where they attach the mandible base; L11, the middle point of the posterior margin of labrum; L12, the apical tooth of the mandible. The data underlying this Figure may be found at [https://doi.org/10.5061/dryad.d7wm37q0t] [41].
(PDF)

S11 Fig The mandible and labrum anatomy of GRP and L-TRAP *Strumigenys* species. (A) Mandibles and labrum of *Strumigenys margaritae*, a typical GRP *Strumigenys*. (B) Mandibles and labrum of *Strumigenys cacaoensis*, a typical L-TRAP *Strumigenys*. The dashed line in light blue illustrates the measurement of the “latch angle,” with lines drawn through the base of the apical tooth and the basal mandibular process of each mandible. abl, articulatory border of labrum; bm, basal margin; bmp, basal mandibular process/lamella; bpl, basal process pocket of labrum; dg, diastemmic gap; em, external margin; lbl, labral lobe/labral glossae; lbr, labrum; llp, lateral labral pocket; mcl, medium cleft of labrum; md, mandible; mda, basal border of mandibular articulations including dorsal and ventral musculature attachment swellings; mm, masticatory margin; msr, mechanosensory receptors.
(PDF)
