# Supplementary material for: Functional innovation promotes diversification of form in the evolution of an ultrafast trap-jaw mechanism in ants
Source: PLoS Biol. 2021 Mar 2;19(3):e3001031. doi: 10.1371/journal.pbio.3001031 (PMC7924744; doi:10.1371/journal.pbio.3001031)
Supplement: S1 Text — (PDF) [file pbio.3001031.s005.pdf]

S1 Text: Further background, analysis, and discussion of *Strumigenys* mandible morphology in the context of the evolution of trap-jaw mechanisms.

Background on morphology of the ant mandible system relevant for understanding the evolution of the trap mechanism in *Strumigenys.*

The plesiomorphic condition in ants is a pair of triangular mandibles each with an external margin, an inner basal margin, and a masticatory margin with a serial row of teeth (S11 Fig). The mandibles move primarily in a planar back-and-forth motion and are attached to two sets of associated muscles; one set of muscles opens the mandibles while the other set closes them [1]. Below the mandibles is the labrum, a flap that articulates with the distal clypeal margin at the anterior portion of the head in a hinge-like up/down movement operating to close the buccal cavity [1-3]. The labrum is typically bilobed with a medial cleft; the anterior margins of these lobes are variously shaped and often have setae present [3]. A single set of muscles closes of the labrum. The mandibles and labrum open and close independently, with no contact, latch, or locking mechanism. The labrum is thought to mainly protect the mouthparts but may have other sensory or mechanical functions.

The muscles responsible for mandible opening and closing are attached to the mandibles indirectly via apodeme filaments (filamentous unsclerotized cutaneous tissues analogous to vertebrate ligaments) or apodemes (sclerotized cutaneous tissues analogous to vertebrate tendons). In ants, the closing muscles are the most prominent muscle in the head capsule and are typically composed of both fast- and slow-twitch muscle fibers [4]. Slow-twitch muscle fibers comprise the bulk of the closing muscle and have relatively short sarcomeres, are attached to the posterior and lateral sides, and are connected to each other via apodeme filaments at more oblique angles. These apodeme filaments in turn connect to the apodeme of the mandible. Fast-twitch muscle fibers are present as a wide, flattened, central band that runs directly from the apodeme of the mandibles to the posterior margin of the head [5]. These fast-twitch muscles have a higher contraction velocity which is important for the quick mandible closure (absent a trap mechanism) for effective prey capture. The slow-twitch muscles provide the more delicate mandible movements used for nest building, grooming, and other fine motor activities [4-6]. Neither the opening muscles of the mandibles nor the labrum closing musculature in *Strumigenys* are known to be different from those of typical ants and are omitted from generalized ant comparisons.

Pre-adaptations and initial steps toward trap-jaw morphotypes

There are two important morphological differences between gripping-type (GRP) *Strumigenys* and other generalized myrmicine ants that may have represented pre-adaptations to evolving the trap mechanism and promoted specialization on Collembola prey: 1) mechanoreceptors that look like elongate hairs extend anteriorly from the labrum and are often referred to as trigger-hairs [7, 8] and 2) a thin flattened "basal process" of the mandible that projects outward at the junction of the inner basal margin and the masticatory margin [9] (Fig 5, msr, bm, S11 Fig). The mechanoreceptors present at the tips of the labrum in GRP *Strumigenys* resemble a short and loosely bristled fine tipped brush. In L-TRAP species these sensors resemble a pair of elongate hairs. In many of the S-TRAP species, these hair-like sensors remain short, fine, and more numerous, interspersed with other specialized setae along a flattened anterior labral border. These hairlike sensors act to trigger both labrum and mandible closure simultaneously through neurons that connect and co-activate them [6, 7]. The second important plesiomorphic character of *Strumigenys* is the basal mandibular process (located at the junction of the inner basal margin and masticatory margin). This process is usually larger and different in shape than the following teeth and has no apparent masticatory function. This process, although shared with other distantly related genera (*Stegomyrmex, Tatuidris, Calyptomyrmex,* and *Octostruma*), is likely independently derived in *Strumigenys*, as it is unlike the basal tooth present at this junction in the two genera forming the sister-clade of *Strumigenys* (*Phalacromyrmex* and *Pilotrochus* [10, 11]). Though the initial function of this process is not known, it may serve to protect the sensitive sensory hairs and labral lobes from damage that could be caused by the power-amplified springing action of Collembola during predation, or damage caused by struggling prey after gripping.

The power-amplified jumping behavior of *Strumigenys* prey likely selectively promoted evolution of the trap-jaw mechanism. Power amplification is likely to increase the success of prey capture as it can close mandibles faster than the spring-loaded release mechanism can power the escape of their prey [7, 9, 12]. When hunting, *Strumigenys* have mandibles held (GRP) or locked (PAM) open with labrum hinged upward and sensory hairs extending from the tips of the labrum in the direction of prey (S1-S3 Movies, S11 Fig). These sensory hairs trigger mandible closure when touched (S2 Movie). In open position with sensory hairs pointed forward, the labrum is in the same plane or nearly in the same plane as the mandibles they close. In GRP species, the labrum arcs downward just prior to the mandible closure but after mandible closure has been initiated (form 1 in Movie S3), whereas in trap-jaw species the labrum is pulled down to release the basal mandibular process to initiate mandible closure (Fig 5, forms 2-8 in S3 Movie). Selection acting on ability to sense prey will influence the shape and length of the labrum while also acting on the mechanics of the labrum and mandibles. It is easy to construct a scenario wherein

contact between the mandibular process and the labrum in GRP species occurred and therefore interfered with mandible closure. This interference could stop mandible movement or alternatively provide a trap-like snapping motion. This initial contact could provide the selection potential for the evolution of latch mediated spring actuation (LaMSA).

We propose that the first step in the evolution of LaMSA is the evolution of a primitive locking mechanism, where the basal process of GRP is modified so that fits into a complementary modified notch on the side of the labrum (form 2 in Movie S3). In this locked position energy can be stored in closing muscles until the labrum is pulled down, releasing the basal process from the pocket (Movie S3). This stored elastic energy powers mandible closure at speeds unachievable by muscle contraction alone [7]. In initial S-TRAP states, the basal process and labrum are modified such that they begin to contact and latch, but only when the mandibles are barely open and nearly parallel. In this state the mandible and labrum shapes are almost indistinguishable from GRP species. Additionally, S-TRAP species derived from GRP show various intermediate states, culminating in the most extreme L-TRAP species, where the basal process is located at the very base of the mandible and the labrum is very short and T-shaped, allowing latch angles of 180 degrees or larger. The location of the locking mechanism can migrate toward anterior or posterior of the mandibles, permitting transitions from S-TRAP to L-TRAP, and vice versa (Movie S3).

Detailed morphological changes associated with the evolution of trap-jaw mandibles

Morphological changes to muscles (Figs. 1, 2, 4, S4 Movie): Muscles with fibers that attach in a more "pennate" or oblique angle to the direction of action generally maximize force, whereas fibers that attach more parallel to the direction of action produce faster movements, thus comparing this property gives some information about mandible muscle performance in ants [4]. In GRP *Strumigenys*, the closing muscles are comprised almost entirely of muscle fibers in a "fast" orientation along the long axis of the head more in line with the direction of action on the mandible, a stark difference from the typical ground plan of ants which are dominated by "slow" fiber orientation [4], and our performance data show that GRP *Strumigenys* have the fastest mandible closure of any measured ant species without power amplification mechanisms (Fig 3, Movie S2, data on other ants from ref. [4]). The closing muscles change from primarily fast orientation in GRP species to almost exclusively slow muscle fibers in more derived S-TRAP and all L-TRAP species (based on attachment orientation [5, 7]. In the ancestral GRP condition,

the closing muscle attaches at or below the midline of the head. As more slow muscle fibers are gained in more derived TRAP morphs, the muscle enlarges and migrates dorsally and laterally. The end effect is a pair of shortened muscles concentrated in the posterior half of the head, each attached to an elongated apodeme. At one end, the apodeme attaches to muscle bundles via numerous apodeme filaments, and at the opposite end attaches directly to a mandible. As the latching angle increases from S-TRAP to L-TRAP, the evolution of apodeme elongation allows it to wrap around the base of the mandible without pinching muscle fibers and visually resembling the initial coil of a catapult. Although the

Morphological changes to mandibles (Figs. 1, 2, 4, Movies S3, S4): In the primitive condition, the basal process is lamellate, projects over the labral lobes, and provides protection to the labrum when the mandibles are closed [9, 11]. In trap jaw species, this process shifts basally and thickens into a process that takes various shapes. In S-TRAP derived from GRP, the process is most often described as approximately trapezoidal in shape, narrowing toward their apex and directed at nearly a 90° angle from the base of the mandible at the point from which it arises. The process remains flattened but enlarges and becomes more sclerotized, having a truncated apical margin that latches into a complementary shaped lateral labral pocket. Similar to GRP, the teeth tend to interlock along their masticatory margins. In L-TRAP species, these interlocking teeth are reduced to denticles or are entirely absent, having only two to a few teeth that overlap only at the apex of the mandible. The basal process in L-TRAP is also somewhat variable but can be generally described as spiniform to cylindrical-elongate in shape. This process arises near the base of the mandible, curving sharply at the base from which it arises, so that it is directed toward the posterior of the head when mandibles are closed and runs parallel with the inner margin of mandible. The basal placement, length, and direction of this process, together with a relatively wider labrum, promote a wider opening angle of greater than 250° in many L-TRAP species —S-TRAP forms typically opening between 18°–90° (mean 64.3°, S.E.: 6.7°, n=60) and L-PAM typically between 53°–215° (mean 131°, S.E.: 7.6°, n=132, ANOVA, $F(1,190)$: 192.1, $p<0.0001$). In S-TRAP derived from L-TRAP, the shape and orientation of the process typically remains similar to L-TRAP, but in extreme S-TRAP reversions where the latching angle is reduced to less than 90° (*e.g. S. anderseni*), the process becomes more similar to primitive S-TRAP derived from GRP. In S-TRAP derived from L-TRAP, the mandible shortens, the relative edentate gap between the clypeus and apical teeth decreases, and the number of teeth increase. In the most extreme reversions, these teeth interlock and are not seemingly different from many GRP species.

Morphological changes of the labrum (Figs. 1, 2, 4, S10, Movies S3-S4): The first noticeable labrum changes appearing in early S-TRAP *Strumigenys* are the development of a complementary pocket to the basal mandibular process, the sclerotization and fusion of the medial cleft, and the elongation of apical hairlike mechanosensors. In the primitive GRP *Strumigenys*, the basal portion of the labrum is broadest, having heavily sclerotized lateral cornulae without indentions or pockets present along the lateral border. The labrum is bilobed, divided into lateral halves by a deeply incised medial cleft which forms labral glossae that continue to freely extend apically and from which a small hair or tuft of hairs usually extend. Together these characters give the labrum a typical GRP a triangular shape [2]. In early trap-jaw species, lateral labral pockets migrate or develop closer to a point lateral to the base of the labral lobes, giving this portion of the labrum a more quadrate shape. The glossae become lobelike and sensory hairs elongate. In more derived S-TRAP and L-TRAP species, the labral pockets migrate basally (as the basal mandibular process also migrates basally) and the labral lobes are often not much more than minute lobes from which a pair of elongated sensory hairs extend. The shape in highly derived trap-jaw species becomes quadrate to broadly rectangular, with extremely shallow to flat anterior lobes sometimes described as T-shaped (Fig 4, S11, forms 5-7 in S3 Movie). As the basal process becomes more elongate in more derived trap-jaw species, a baso-dorsal pocket evolves to house the processes when the labrum is closed. This pocket-like structure is not known to occur in any other ants and yet is present in each evolution of LaMSA. This pocket presumably has a dual function: to keep the mandibles from hyper extending, and to keep the basal processes from puncturing themselves during rapid closure.

Morphological changes in head shape (Movies S3, S4): All of the changes associated with the trap-jaw mechanism (mandible, labrum, and internal head musculature) are associated with predictable changes in head shape. The clypeus, in primitive GRP condition, is a large triangular shield fused along the posterior two sides of the head and with a free anterior margin that extends forward to cover the basal portion of the mandibles. The mandibles articulate underneath the clypeus and are limited in their opening angle by where the lateral borders of the clypeus and head join. In early S-TRAP species and a few S-TRAP species derived from L-TRAP, the clypeus is similarly large and shaped like GRP species. In most S-TRAP and L-TRAP species, the clypeus reduces in size to a small, unshielded triangle that exposes the basal portion of the mandible and does not restrict the opening angle. In the most derived L-TRAP species, the mandible opens to 270° and when locking open comes into contact with the ocular groove (a groove running between the dorsal and ventral surfaces from the eye to the base of mandible).

The reduction in size of the mandible is coupled with the expanded frontal lobes needed to house enlarged slow-twitch and more powerful musculature, making the head of L-TRAP species heart-shaped. A similarly striking convergence in head shape occurs across the three independent L-TRAP clades.

1. Gotwald WH, Jr. Comparative morphological studies of the ants, with particular reference to the mouthparts. Cornell University Agricultural Experiment Station Memoir. 1969;408:1-50.
2. Brown WL, Wilson EO. The evolution of the dacetine ants. Q Rev Biol. 1959;34(4):278-94.
3. Richter A, Keller RA, Rosumek FB, Economo EP, Garcia FH, Beutel RG. The cephalic anatomy of workers of the ant species *Wasmannia affinis* (Formicidae, Hymenoptera, Insecta) and its evolutionary implications. Arthropod Struct Dev. 2019;49:26-49.
4. Gronenberg W, Paul J, Just S, Holldobler B. Mandible muscle fibers in ants: Fast or powerful? Cell Tissue Res. 1997;289(2):347-61.
5. Paul J, Gronenberg W. Optimizing force and velocity: Mandible muscle fibre attachments in ants. J Exp Biol. 1999;202(7):797-808.
6. Paul J, Gronenberg W. Motor control of the mandible closer muscle in ants. J Insect Physiol. 2002;48(2):255-67.
7. Gronenberg W. The trap-jaw mechanism in the dacetine ants *Daceton armigerum* and *Strumigenys* sp. J Exp Biol. 1996;199(9):2021-33.
8. Forel A. Les fourmis de la Suisse. Soc Helvetique Sci Natur Mem. 1874;26:452.
9. Bolton B. Ant genera of the tribe Dacetonini (Hymenoptera : Formicidae). J Nat Hist. 1999;33(11):1639-89.
10. Ward PS, Brady SG, Fisher BL, Schultz TR. The evolution of myrmicine ants: phylogeny and biogeography of a hyperdiverse ant clade (Hymenoptera: Formicidae). Systematic Entomology. 2015;40(1):61-81.
11. Baroni Urbani CDA, M.L. The ant tribe Dacetini: limits and constituent genera, with descriptions of new species. Annali del Museo Civico di Storia Naturale “G Doria”. 2007;99:1-191.
12. Christian E. Jump of Springtails. Naturwissenschaften. 1978;65(9):495-6.
