# Supplementary material for: Functional innovation promotes diversification of form in the evolution of an ultrafast trap-jaw mechanism in ants
Source: PLoS Biol. 2021 Mar 2;19(3):e3001031. doi: 10.1371/journal.pbio.3001031 (PMC7924744; doi:10.1371/journal.pbio.3001031)
Supplement: S2 Text — (PDF) [file pbio.3001031.s006.pdf]

S2 Text: Further detail about the number of independent evolutions of Trap-Jaw Mandibles.

We used ancestral state estimation to infer the evolutionary history of mandible design in *Strumigenys*, with a focus on the evolution of power amplification as a discrete, binary trait. As described in the methods, we used a combination of time homogeneous models assuming symmetric (Mk1) and asymmetric (Mk2) transition rates, as well as a Bayesian analysis of time-varying Mk2 models with a Random Local Clock model that allows for the possibility that the trait evolves at different rates in different parts of the tree. As a secondary analysis, mostly for visualization purposes, we modeled the evolution of the three ecomorphs (GRP, S-TRAP, L-TRAP) although the distinction between S-TRAP and L-TRAP is more continuous than the distinction between GRP and trap-jaw mandibles.

In general, all the models agreed that the gripping mandible is most probably ancestral in the genus, followed by gains of the trap mechanism (e.g. latch mediated spring actuation, LaMSA) within the clade (Figs. 2, S1, S2). Although outgroups were not present in the ancestral state analysis, all close relatives (including the sister lineage, a clade consisting of the genera *Pilotrochus* and *Phalacromyrmex*) of *Strumigenys* have non-LaMSA mandibles. Thus, if LaMSA was the ancestral state of the genus followed by losses, the gain event would have had to occur on the stem lineage. Our analysis made the conservative assumption of assuming an equal prior on the crown node of *Strumigenys*, but still consistently recovered gripping as ancestral (state of the *Strumigenys* crown node) with LaMSA evolving within the radiation.

However, our opinion is that some uncertainty remains regarding precisely how many gains occurred and whether there were any losses. This is particularly true in the Neotropical clade. Across the rest of the tree, the pattern of gains is very stable across models because the trap mechanism is either present or absent across large clades. However, in the Neotropical clade, the GRP and TRAP are more intermingled with each other on the tree, especially the short-mandibled trap-jaw clades (including the *excisa, paradoxa, nitens, appretiata, beebei,* and *hyphata* groups). The symmetric models typically model this as several gains and several losses (with around 7 gains and 2 total losses across the genus). In contrast, the asymmetric models typically model 10 gains and no losses, the minimum number to explain the tip states without any losses. The asymmetric model generally disfavors losses because the rest of the tree contains not a single case of a reversion, even within gigantic trap-jaw clades. Although the asymmetric models are strongly favored (ΔAIC of ~18, for both topologies), there is of course no guarantee that the maximum likelihood model is correct. Relatively minor phylogenetic rearrangements within the Neotropical clade, or additions of new taxa to different parts of that clade, could tip the balance

between gains and losses in that branch of the tree. This is why, to be conservative, we report 7–10 gains as our main conclusion even though the models favor 10 gains. Further taxon sampling in the S-TRAP Neotropical groups is needed to make more confident inferences on the precise pattern of gains and losses. However, the difference between 7–10 is only relevant for understanding the detailed history of the Neotropical clade, and does not affect the overall conclusion of our study that power-amplification has been gained many times in the genus. The latter is a consistent finding of all our analyses.
