# Supplementary material for: Functional innovation promotes diversification of form in the evolution of an ultrafast trap-jaw mechanism in ants
Source: PLoS Biol. 2021 Mar 2;19(3):e3001031. doi: 10.1371/journal.pbio.3001031 (PMC7924744; doi:10.1371/journal.pbio.3001031)
Supplement: S1 Fig — Strumigenys is a pantropical, hyperdiverse genus of leaf litter predators whose preferred prey are usually (A) springtails—leaf-litter arthropods with a power-amplified spring-like escape mechanism (furculum). Traditionally, Strumigenys have been divided into 2 main ecomorphs [23]: (B) short-mandibled forms, that tend to be more cryptobiotic and subterranean feeders that employ a strategy of luring or cautiously approaching prey, then gripping onto and stinging the struggling prey item, and (C) long-mandibled forms that use the trap-jaw mechanism to strike and stun, lift, then sting their prey, and are more active hunters (also see S1 Movie). We also report here that several groups of short-mandibled forms actually have a trap-mechanism. The drawings are by Mayuko Suwabe. (PDF) [file pbio.3001031.s007.pdf]

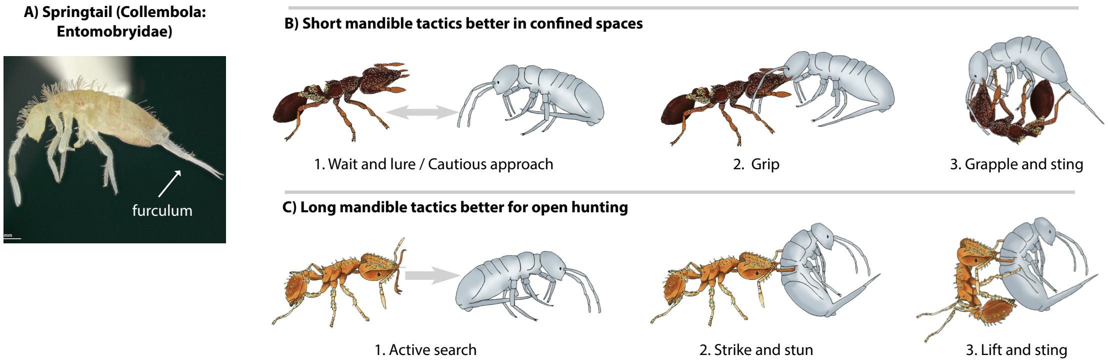

Figure S1. | Hunting tactics of short and long-mandibled *Strumigenys* ants. *Strumigenys* is a pantropical, hyperdiverse genus of leaf litter predators whose preferred prey are usually a) springtails—leaf-litter arthropods with a power-amplified spring-like escape mechanism (furculum). Traditionally, *Strumigenys* have been divided into two main ecomorphs [23]: b) short mandibled forms, that tend to be more cryptobiotic and subterranean feeders that employ a strategy of luring or cautiously approaching prey, then gripping onto and stinging the struggling prey item, and c) long-mandibled forms that use the trap-jaw mechanism to strike and stun, lift, then sting their prey, and are more active hunters (also see S1 Movie). We also report here that several groups of short-mandibled forms actually have a trap-mechanism. The drawings are by Mayuko Suwabe.
