# Supplementary material for: Functional innovation promotes diversification of form in the evolution of an ultrafast trap-jaw mechanism in ants
Source: PLoS Biol. 2021 Mar 2;19(3):e3001031. doi: 10.1371/journal.pbio.3001031 (PMC7924744; doi:10.1371/journal.pbio.3001031)
Supplement: S2 Fig — Ancestral state probabilities under (A) the time-homogeneous symmetric (Mk1) model, (B) the time-homogeneous asymmetric (Mk2) models are marginal probabilities of node states from 500 stochastic character maps using the maximum likelihood transition matrix. (C) Results of a Bayesian MCMC analysis of the asymmetric (Mk2) model allowing for rate changes on the tree, implemented in BEAST. In each case, ancestral state probabilities (marginal or posterior) were calculated for each node, then branchwise state probabilities were visualized using the contMap function in R. (D) The maximum likelihood (for Mk1 and Mk2) or posterior means (for Mk2-RLC) for each parameter or inferred number of transitions/rate changes. (PDF) [file pbio.3001031.s008.pdf]

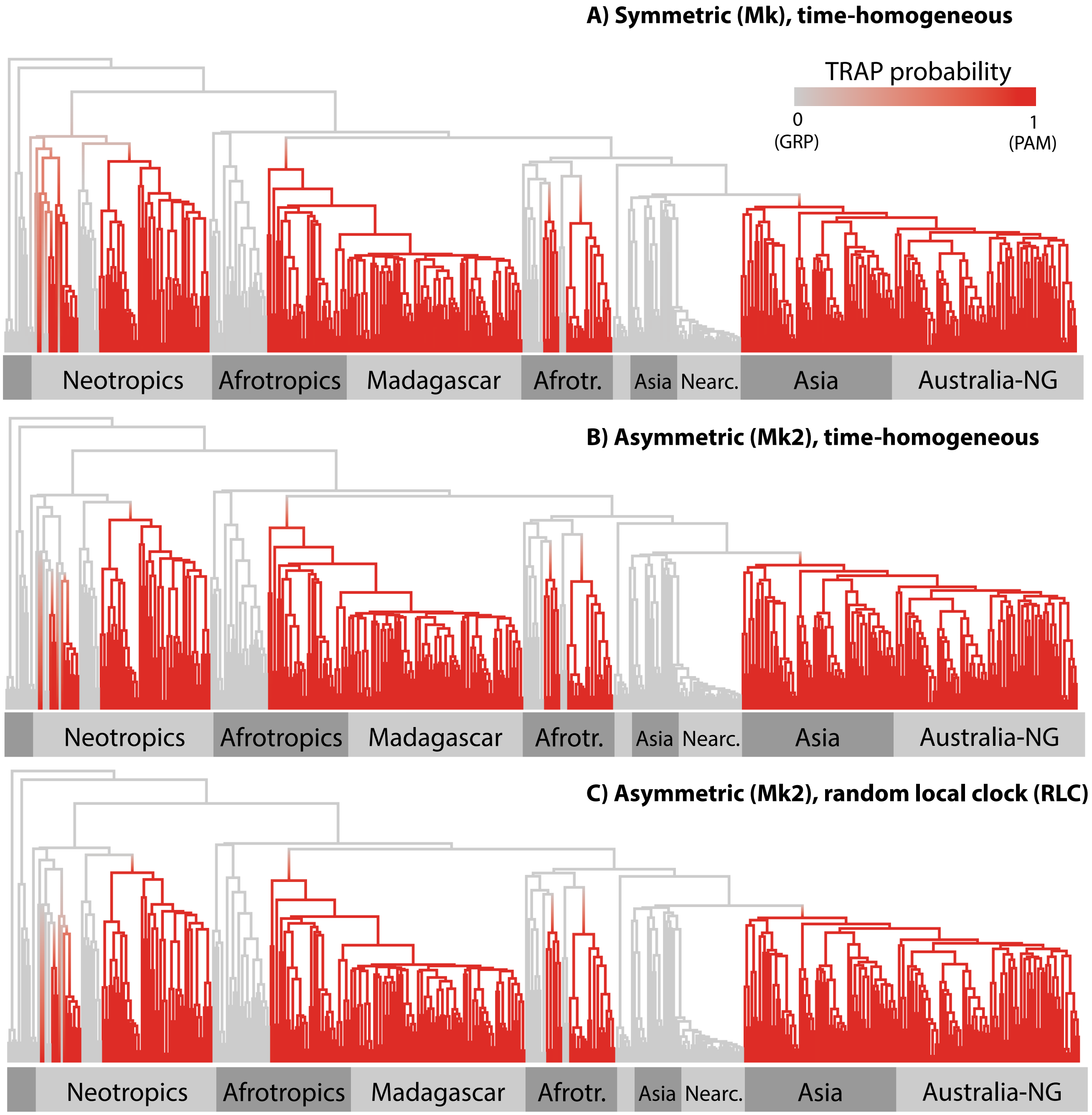

Fig. S2. | Ancestral state reconstruction of the presence or absence of trap-jaw mandibles (TRAP) under different models of character evolution under rooting 1. Ancestral state probabilities under A) the time-homogeneous symmetric (Mk1) model, B) the time-homogeneous asymmetric (Mk2) models are marginal probabilities of node states from 500 stochastic character maps using the maximum likelihood transition matrix. C) Results of a Bayesian MCMC analysis of the asymmetric (Mk2) model allowing for rate changes on the tree, implemented in BEAST. In each case, ancestral state probabilities (marginal or posterior) were calculated for each node, then branch-wise state probabilities were visualized using the contMap function in R. D) The maximum likelihood (for Mk1 and Mk2) or posterior means (for Mk2-RLC) for each parameter or inferred number of transitions/rate changes.

D)

Model	GRP ⟶ TRAP rate	GRP ⟶ TRAP count	TRAP⟶GRP rate	TRAP⟶GRP count	rate changes	ΔAICc
Mk1	0.0027	7.8	0.0027	2.7	n.a.	17.9
Mk2	0.0092	10.7	0.0000	0.0	n.a.	0.0
Mk2-RLC	0.0074	10.4	0.0004	0.2	0.59	n.a.
