# Supplementary material for: Functional innovation promotes diversification of form in the evolution of an ultrafast trap-jaw mechanism in ants
Source: PLoS Biol. 2021 Mar 2;19(3):e3001031. doi: 10.1371/journal.pbio.3001031 (PMC7924744; doi:10.1371/journal.pbio.3001031)
Supplement: S4 Fig — The tree, including 132 taxa was inferred with ExaML. Node supports reflect bootstrap (left) and booster (right) scores from 100 bootstraps. The tips are annotated with species name, ecomorph (GRP, S-TRAP, L-TRAP), latch angle, and geographic region. The bars on the right reflect the dominant geographic region for each clade, although some lineages within the clade may be in different regions. The red dot shows the placement of the ambatrix and capitata groups sister to the neotropical clade (we call this “root 2” position). The alternative rooting of the Strumigenys clade has ambatrix and capitata sister to the rest of the Strumigenys (see S5 Fig) and was inferred in the full analysis, although neither placement is well supported in the 2 analyses. (PDF) [file pbio.3001031.s010.pdf]

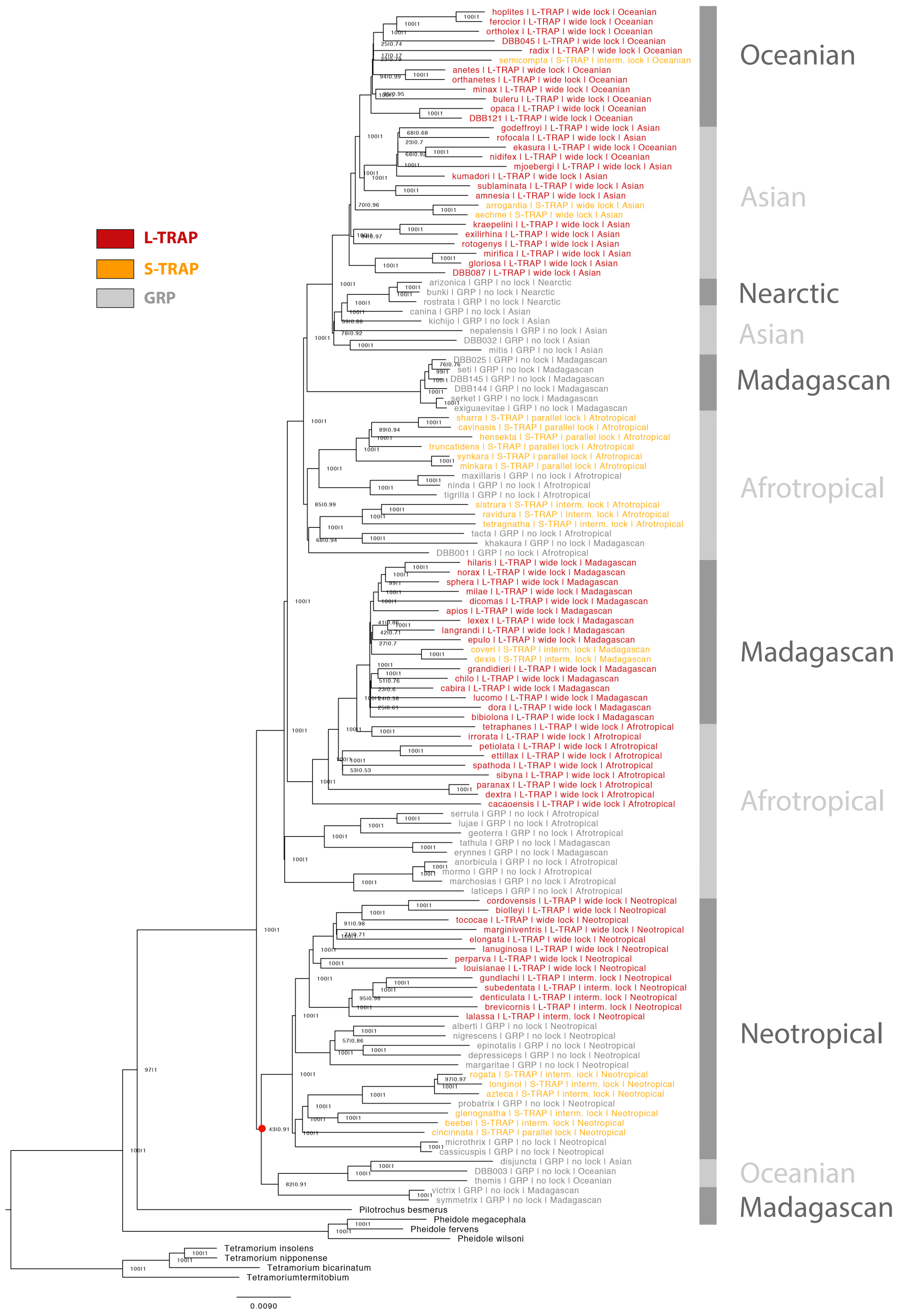

Fig. S4. | Maximum Likelihood backbone phylogeny of the ant genus *Strumigenys* using only species with the highest data coverage to represent individual clades. The tree including 132 taxa was inferred with ExaML. Node supports reflect bootstrap (left) and booster (right) scores from 100 bootstraps. The tips are annotated with species name, ecomorph (GRP, S-TRAP, L-TRAP), latch angle, and geographic region. The bars on the right reflect the dominant geographic region for each clade, although some lineages within the clade may be in different regions. The red dot shows the placement of the *ambatrix* and *capitata* groups sister to the neotropical clade (we call this "root 2" position). The alternative rooting of the *Strumigenys* clade has *ambatrix* and *capitata* sister to the rest of the *Strumigenys* (see S5 Fig.) and was inferred in the full analysis, although neither placement is well supported in the two analyses.
