# Supplementary figures and images for: Functional innovation promotes diversification of form in the evolution of an ultrafast trap-jaw mechanism in ants
Source: PLoS Biol. 2021 Mar 2;19(3):e3001031. doi: 10.1371/journal.pbio.3001031 (PMC7924744; doi:10.1371/journal.pbio.3001031)

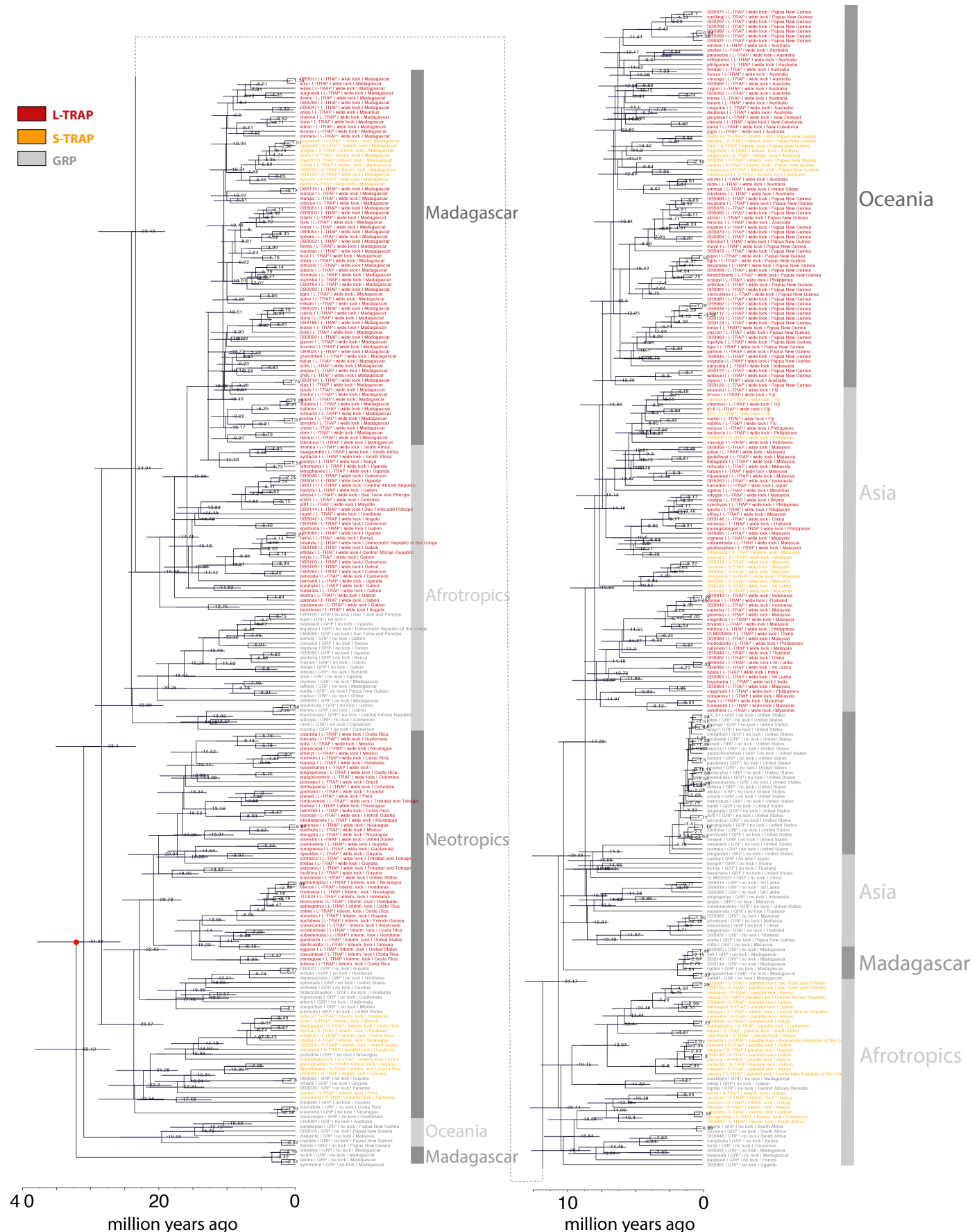

Supplement: S5 Fig — Nodes are annotated with common ancestor ages and 95% height ranges from a posterior sample of a Bayesian analysis. The red dot indicates the position of the ambatrix and capitata groups in “root 1” position. This is the tree used in the analyses, although we also ran analyses using an alternate topology where the ambatrix and capitata groups (bottom left) are sister to the neotropical clade (”root 2” position, see S3 Fig). (PDF) [file pbio.3001031.s011.pdf]
