# Supplementary material for: Functional innovation promotes diversification of form in the evolution of an ultrafast trap-jaw mechanism in ants
Source: PLoS Biol. 2021 Mar 2;19(3):e3001031. doi: 10.1371/journal.pbio.3001031 (PMC7924744; doi:10.1371/journal.pbio.3001031)
Supplement: S6 Fig — Nodes are annotated with bootstrap and booster scores based on 100 bootstraps. (PDF) [file pbio.3001031.s012.pdf]

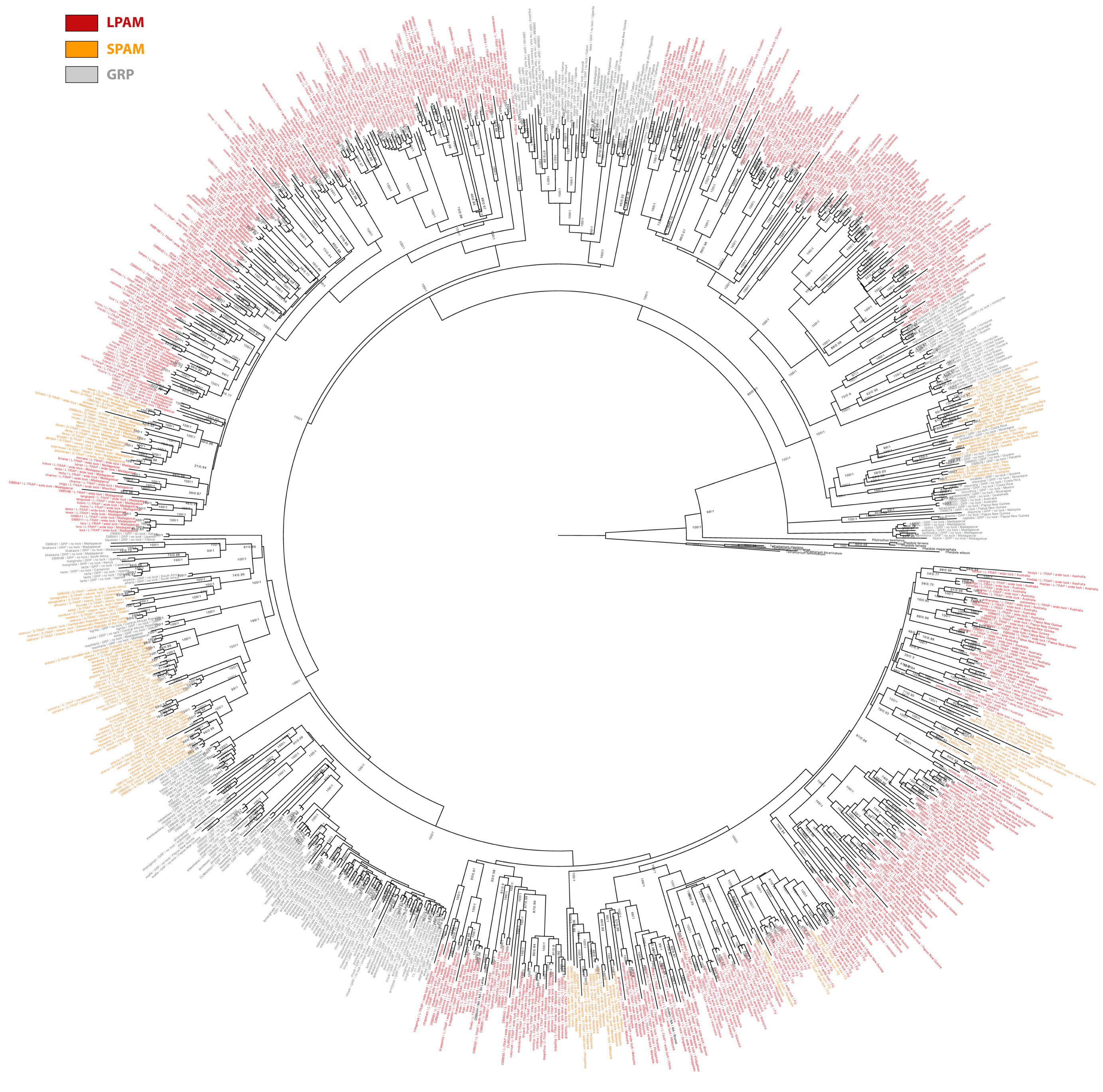

Fig. S6. | The maximum likelihood tree for the full dataset (885 specimens) inferred with ExaML. Nodes are annotated with bootstrap and booster scores based on 100 bootstraps.
