# Supplementary material for: Functional innovation promotes diversification of form in the evolution of an ultrafast trap-jaw mechanism in ants
Source: PLoS Biol. 2021 Mar 2;19(3):e3001031. doi: 10.1371/journal.pbio.3001031 (PMC7924744; doi:10.1371/journal.pbio.3001031)
Supplement: S8 Fig — In RAD-seq phylogenomics, missing data at tips can either be structured by clade (usually due to mutation-disruption) or randomly across the phylogeny (usually due to low sequencing coverage). Eaton and colleagues showed that while the former is a problem for phylogenetics, because of lack of overlapping data among distant clades to inform in deeper relationships, but the latter is less of an issue. Indeed, including more data present in few individuals is beneficial because it can inform deeper nodes. As deeper branches can be informed by more tips, the number of loci potentially expands dramatically because they have more chances to be recovered in descendents of each branch. Here, we plot the number of potentially quartet informative 30 bp loci for each internal branch (positioned on the node to the right of the branch) of the tree depicted in S4 Fig to be potentially quartet-informative, a locus must be present in 1 tip in among the descendents of each of the 4 branches originating from a focal branch (see Fig 1 in Eaton and colleagues). The accumulation of many informative loci for deeper nodes shows that the pattern of missing data is not highly phylogenetically structured and is thus informative for tree inference on this phylogenetic scale. (PDF) [file pbio.3001031.s014.pdf]

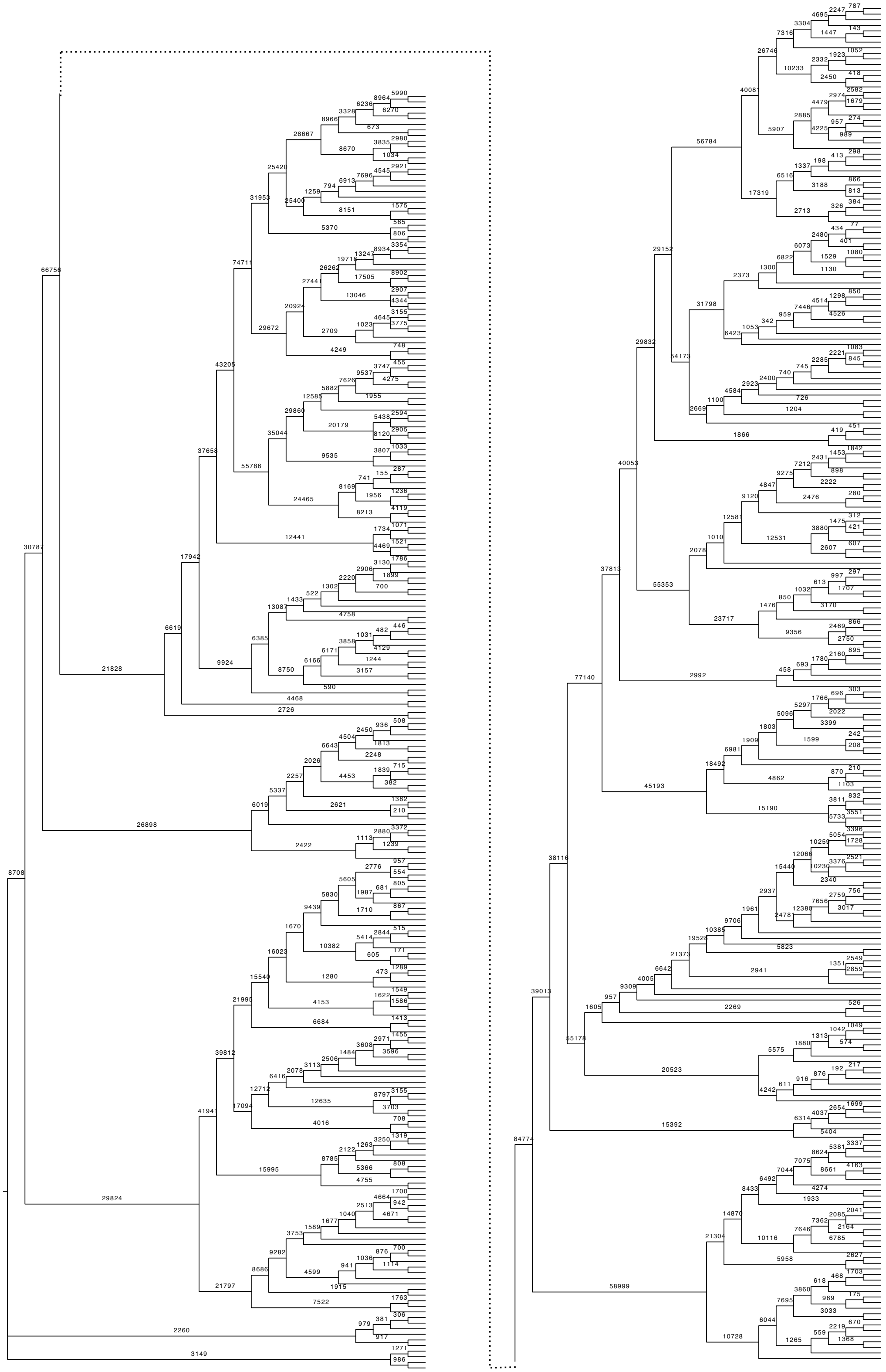

Fig. S8. | The number of potentially quartet-informative loci at each branch. In RAD-seq phylogenomics, missing data at tips can either be structured by clade (usually due to mutation-disruption) or randomly across the phylogeny (usually due to low sequencing coverage). Eaton et al. showed that while the former is a problem for phylogenetics, because of lack of overlapping data among distant clades to inform in deeper relationships, but the latter is less of an issue. Indeed, including more data present in few individuals is beneficial because it can inform deeper nodes. As deeper branches can be informed by more tips, the number of loci potentially expands dramatically because they have more chances to be recovered in descendents of each branch. Here we plot the number of potentially quartet informative 30bp loci for each internal branch (positioned on the node to the right of the branch) of the tree depicted in Fig. S4. To be potentially quartet-informative, a locus must be present in one tip in among the descendents of each of the four branches originating from a focal branch (see Figure 1 in Eaton et al. 2017). The accumulation of many informative loci for deeper nodes shows that the pattern of missing data is not highly phylogenetically structured, and is thus informative for tree inference on this phylogenetic scale.
