# Supplementary material for: Functional innovation promotes diversification of form in the evolution of an ultrafast trap-jaw mechanism in ants
Source: PLoS Biol. 2021 Mar 2;19(3):e3001031. doi: 10.1371/journal.pbio.3001031 (PMC7924744; doi:10.1371/journal.pbio.3001031)
Supplement: S9 Fig — Probabilities of ancestral states in each broad geographic region calculated with stochastic character mapping under a Maximum Likelihood model. The deepest nodes are uncertain, but the main clades within the genus are highly geographically structured. (PDF) [file pbio.3001031.s015.pdf]

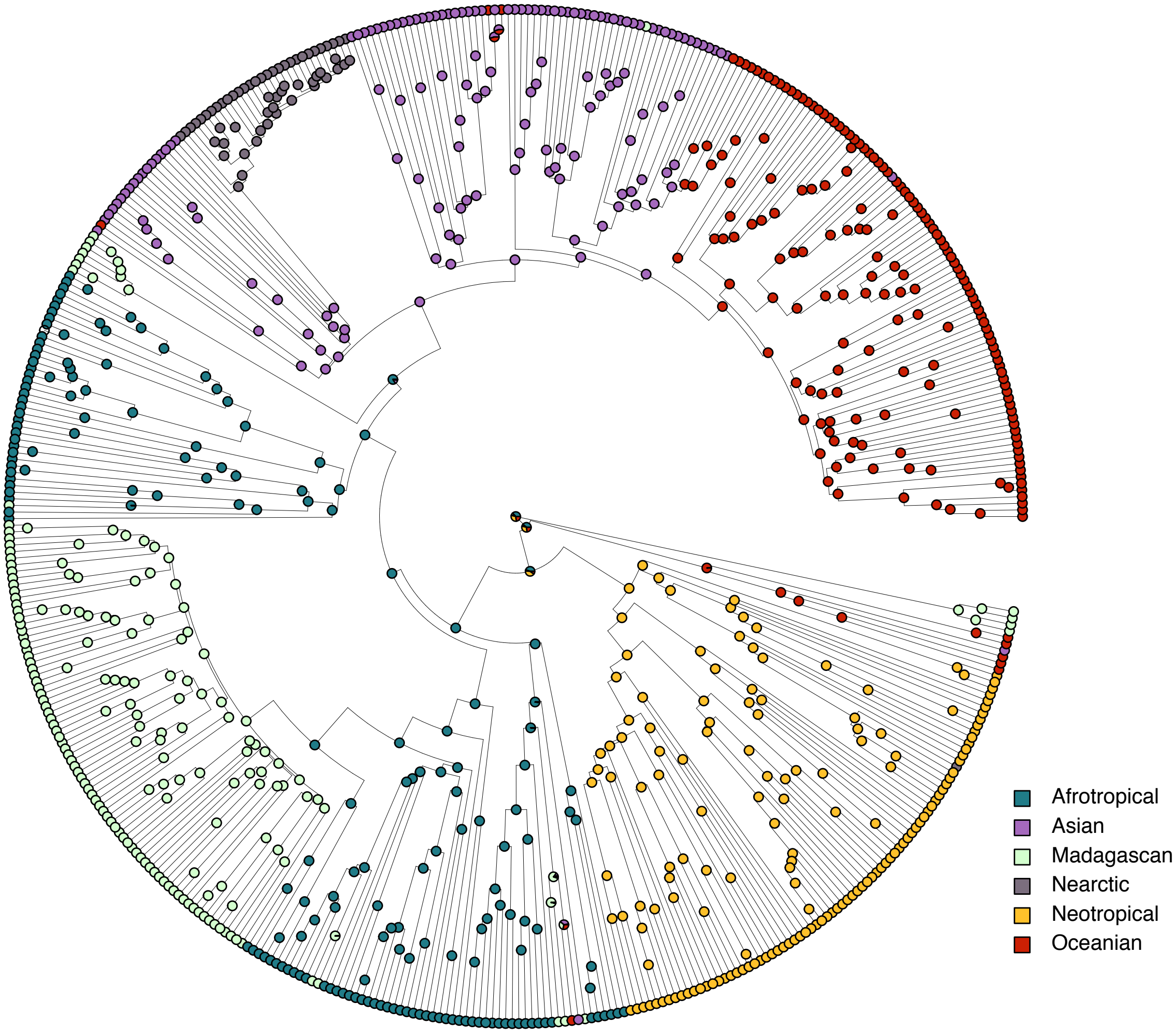

Fig. S9. | Biogeographic history of *Strumigenys*. Probabilities of ancestral states in each broad geographic region calculated with stochastic character mapping under a Maximum Likelihood model. The deepest nodes are uncertain, but the main clades within the genus are highly geographically structured.
