# Supplementary material for: Functional innovation promotes diversification of form in the evolution of an ultrafast trap-jaw mechanism in ants
Source: PLoS Biol. 2021 Mar 2;19(3):e3001031. doi: 10.1371/journal.pbio.3001031 (PMC7924744; doi:10.1371/journal.pbio.3001031)
Supplement: S10 Fig — The inferred PCA morphospace plot for Strumigenys (seen in Fig 5) annotated with species names. Example forms with landmarks placed are below. The landmarks were placed as such: L1–L2, the most posterior points of the closing muscles where they attach to the posterior margin of the head; L3–L4, points on the closing muscles where they attach to the middle point of the posterior margin of the head. L5–L6, the most ventral points of the closing muscles where they attach to the ventral side of the head; L7–L8, the points on the apodeme where the closing muscles start; L9–L10, the most anterior points of closer apodemes where they attach the mandible base; L11, the middle point of the posterior margin of labrum; L12, the apical tooth of the mandible. The data underlying this Figure may be found at [https://doi.org/10.5061/dryad.d7wm37q0t] [41]. (PDF) [file pbio.3001031.s016.pdf]

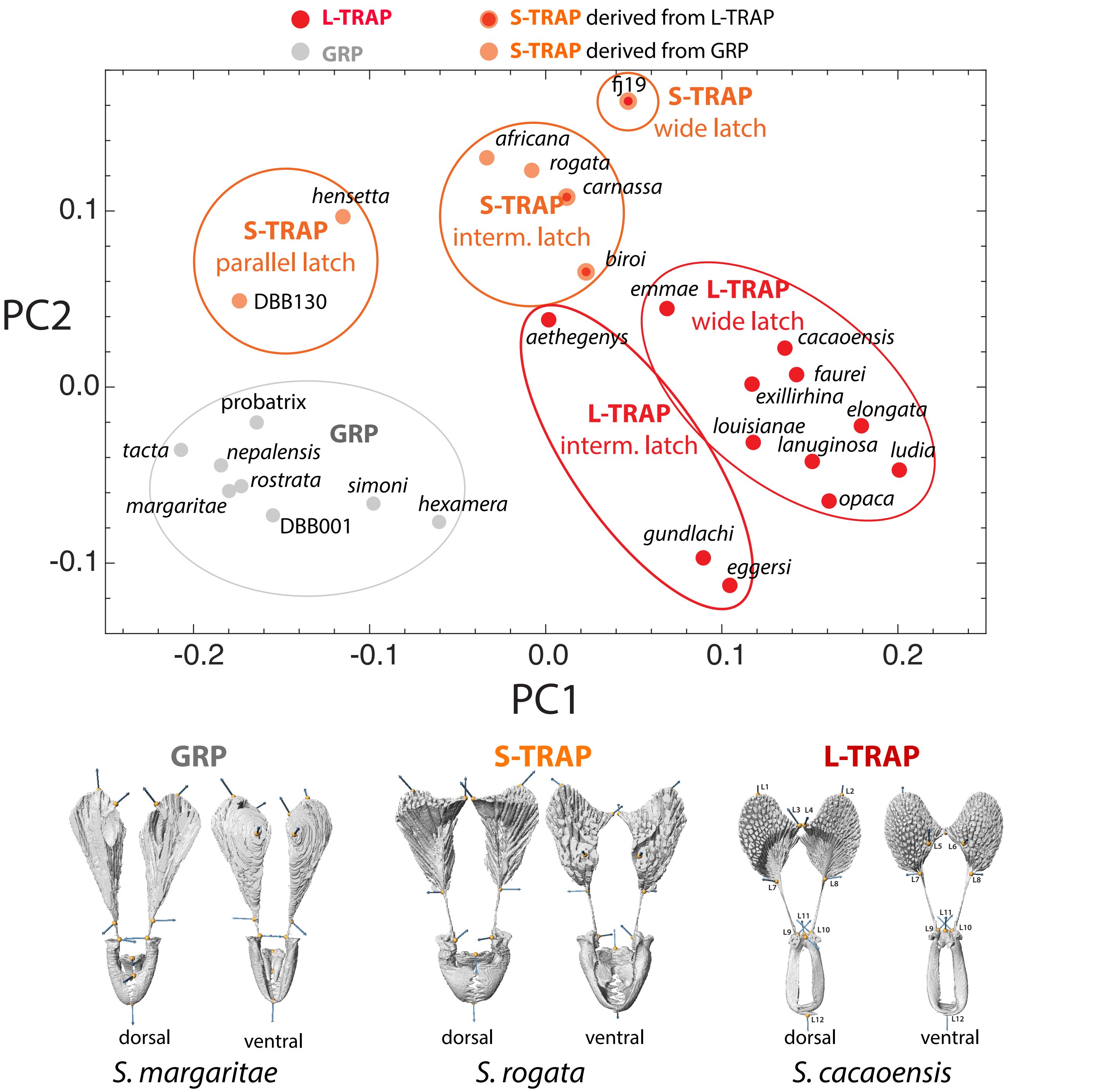

Fig. S10. | 3D morphospace of the mandible system and landmark positions. The inferred PCA morphospace plot for Strumigenys (seen in Fig. 4) annotated with species names. Example forms with landmarks placed are below. The landmarks were placed as such: L1-L2, the most posterior points of the closing muscles where they attach to the posterior margin of the head; L3-L4, points on the closing muscles where they attach to the middle point of the posterior margin of the head. L5-L6, the most ventral points of the closing muscles where they attach to the ventral side of the head; L7-L8, the points on the apodeme where the closing muscles start, L9-L10, the most anterior points of closer apodemes where they attach the mandible base, L11, the middle point of the posterior margin of labrum, L12, the apical tooth of the mandible. The data underlying this Figure may be found at [Dryad doi to be added after acceptance].
