# Supplementary material for: Functional innovation promotes diversification of form in the evolution of an ultrafast trap-jaw mechanism in ants
Source: PLoS Biol. 2021 Mar 2;19(3):e3001031. doi: 10.1371/journal.pbio.3001031 (PMC7924744; doi:10.1371/journal.pbio.3001031)
Supplement: S11 Fig — (A) Mandibles and labrum of Strumigenys margaritae, a typical GRP Strumigenys. (B) Mandibles and labrum of Strumigenys cacaoensis, a typical L-TRAP Strumigenys. The dashed line in light blue illustrates the measurement of the “latch angle,” with lines drawn through the base of the apical tooth and the basal mandibular process of each mandible. abl, articulatory border of labrum; bm, basal margin; bmp, basal mandibular process/lamella; bpl, basal process pocket of labrum; dg, diastemmic gap; em, external margin; lbl, labral lobe/labral glossae; lbr, labrum; llp, lateral labral pocket; mcl, medium cleft of labrum; md, mandible; mda, basal border of mandibular articulations including dorsal and ventral musculature attachment swellings; mm, masticatory margin; msr, mechanosensory receptors. (PDF) [file pbio.3001031.s017.pdf]

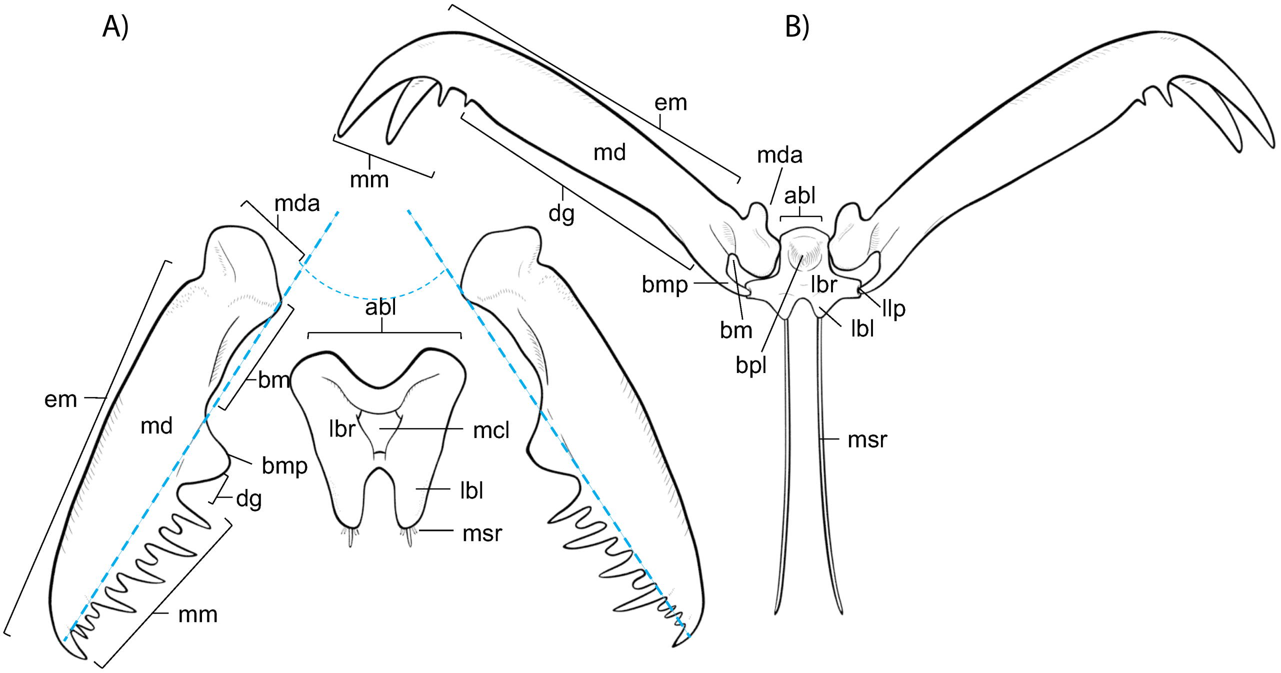

Fig. S11. | The mandible and labrum anatomy of GRP and L-TRAP *Strumigenys* species. A) Mandibles and labrum of *Strumigenys margaritae*, a typical GRP *Strumigenys*. B) Mandibles and labrum of *Strumigenys cacaoensis*, a typical L-TRAP Strumigenys; abl — articulatory border of labrum, bm — basal margin, bmp — basal mandibular process/lamella, bpl — basal process pocket of labrum, dg — diastemmic gap, em — external margin, lbl — labral lobe/labral glossae, lbr — labrum, llp — lateral labral pocket, mcl — medium cleft of labrum, md — mandible, mda — basal border of mandibular articulations including dorsal and ventral musculature attachment swellings, mm — masticatory margin, msr — mechanosensory receptors. The dashed line in light blue illustrates the measurement of the “latch angle”, with lines drawn through the base of the apical tooth and the basal mandibular process of each mandible.
