# Supplementary material for: Functional innovation promotes diversification of form in the evolution of an ultrafast trap-jaw mechanism in ants
Source: PLoS Biol. 2021 Mar 2;19(3):e3001031. doi: 10.1371/journal.pbio.3001031 (PMC7924744; doi:10.1371/journal.pbio.3001031)
Supplement: S7 Fig — To check whether our RAD-seq–based phylogeny was consistent with previous analyses, we compared the topology of subtrees of overlapping taxa between the current analysis (i.e., as seen in S4 Fig) and a previous analyses based on different molecular data (tree from a family wide diversification analysis in Economo and colleagues, most of the data from Ward and colleagues which also inferred a nearly identical topology). In some cases, where the same species was not sequenced, a closely related species from the same species group was substituted for the comparison (S. atopogenys for S. ocypete, S. sistrura for S. olsoni, S. simoni for S. ludovici, S. hubbewatyorum for S. nitens), these are denoted with parentheses. Numbers indicate bootstrap support for nodes that disagreed between the 2 trees. Note the 11-gene phylogeny recovered the ambatrix group in the “root 1” position, sister to the rest of Strumigenys (although capitata group was not included). (PDF) [file pbio.3001031.s013.pdf]

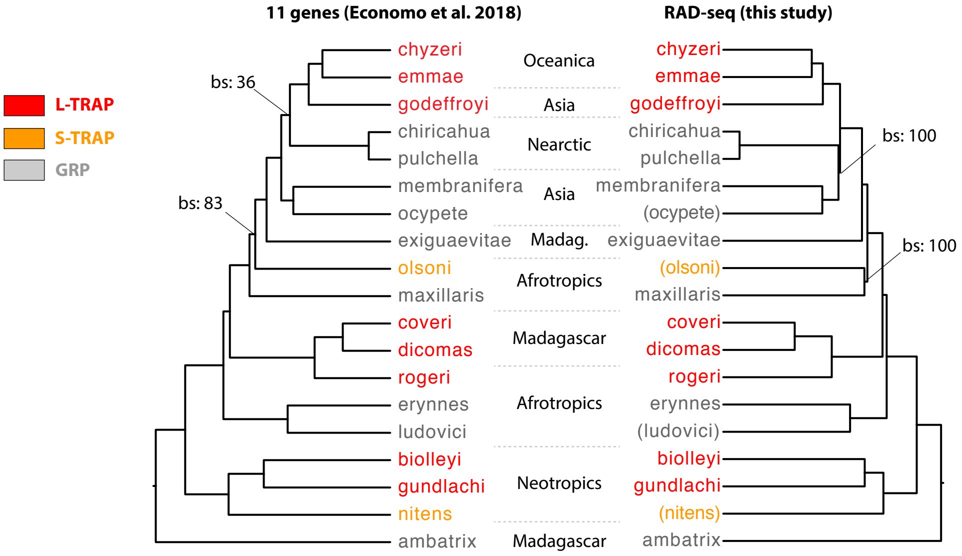

Fig. S7. | Consistency of RAD-seq and gene-based phylogenies. To check whether our RAD-seq based phylogney was consistent with previous analyses, we compared the topology of subtrees of overlapping taxa between the current analysis (i.e. as seen in Fig. S4) and a previous analyses based on different molecular data (tree from a family wide diversification analysis in Economo et al. 2018, most of the data from Ward et al. 2015 which also inferred a nearly identical topology). In some cases, where the same species was not sequenced, a closely related species from the same species group was substituted for the comparison (*S. atopogenys* for *S. ocypete*, *S. sistrura* for *S. olsoni*, *S. simoni* for *S. ludovici*, *S. hubbewatyorum* for *S. nitens*), these are denoted with parentheses. Numbers indicate bootstrap support for nodes that disagreed between the two trees. Note the 11-gene phylogeny recovered the ambatrix group in the "root 1" position, sister to the rest of *Strumigenys* (although *capitata* group was not included).
